# Lateral Hypothalamus Calcium/Calmodulin-Dependent Protein Kinase II *α* Neurons Encode Novelty-Seeking Signals to Promote Predatory Eating

**DOI:** 10.34133/2022/9802382

**Published:** 2022-08-05

**Authors:** Na Tan, Jiaying Shi, Lingyu Xu, Yanrong Zheng, Xia Wang, Nanxi Lai, Zhuowen Fang, Jialu Chen, Yi Wang, Zhong Chen

**Affiliations:** ^1^Institute of Pharmacology and Toxicology, College of Pharmaceutical Sciences, Zhejiang University, Hangzhou, China; ^2^Key Laboratory of Neuropharmacology and Translational Medicine of Zhejiang Province, School of Pharmaceutical Sciences, Zhejiang Chinese Medical University, Hangzhou, China

## Abstract

Predatory hunting is an innate appetite-driven and evolutionarily conserved behavior essential for animal survival, integrating sequential behaviors including searching, pursuit, attack, retrieval, and ultimately consumption. Nevertheless, neural circuits underlying hunting behavior with different features remain largely unexplored. Here, we deciphered a novel function of lateral hypothalamus (LH) calcium/calmodulin-dependent protein kinase II *α* (CaMKII*α*^+^) neurons in hunting behavior and uncovered upstream/downstream circuit basis. LH CaMKII*α*^+^ neurons bidirectionally modulate novelty-seeking behavior, predatory attack, and eating in hunting behavior. LH CaMKII*α*^+^ neurons integrate hunting-related novelty-seeking information from the medial preoptic area (MPOA) and project to the ventral periaqueductal gray (vPAG) to promote predatory eating. Our results demonstrate that LH CaMKII*α*^+^ neurons are the key hub that integrate MPOA-conveyed novelty-seeking signals and encode predatory eating in hunting behavior, which enriched the neuronal substrate of hunting behavior.

## 1. Introduction

Predatory hunting has been regarded as an innate appetite-driven and evolutionarily conserved behavior essential for animal survival [1]. It is a precise action that needs to integrate multiple sensations including vision, audition [2], and tactile information [3] that must be processed to perform sequential behaviors including searching/exploration, chasing, grabbing, biting, retrieval, and consuming [4]. Novelty exploration is the first and important procedure. Both humans and animals retain the novelty-seeking ability to explore unfamiliar stimuli and environments as a natural propensity [5–7] because exploring novel stimuli reduces uncertainty about the environment and is necessary for acquiring information to optimize choice behavior translation into a successful hunting action [8].

Previous research revealed that the periaqueductal gray (PAG) is a key region for executing predatory hunting, integrating upstream prey-related signals. Zona incerta (ZI) GABAergic neurons integrate prey-related visual and whisker signals and encode appetite-driven hunting behavior, through its projection to the PAG [9]. The central amygdala projects to the PAG controlling locomotion during pursuit [10]. Furthermore, PAG-projecting LH GABAergic neurons activation rapidly drove predatory attack [11], and PAG-projecting LH Gad2^+^ GABAergic neurons encoded predatory hunting [12]; however, this GABAergic circuit encodes only predatory hunting rather than prey consumption. Interestingly, neurons in the PAG are largely heterogeneous and mediate both approach and defensive behaviors, indicating that this circuit may encode predation through suppressing defensive responses to prey, which provides us a new perspective toward complex and diverse predatory hunting [12]. Recently, the MPOA-PAG circuit was found to be involved in both novel object exploration and predatory attack without prey consumption in hunting behaviors [13]. However, how novelty-seeking information is integrated into predatory eating in hunting behavior is not fully understood. Here, using Ca^2+^ fiber photometry, optogenetics, chemogenetics, and behavioral tests, we reveal that LH CaMKII*α*^+^ neurons act as a hub that receive the MPOA-conveyed novelty-seeking information and integrate it into predatory eating in hunting behavior through projections to the vPAG, demonstrating a new perspective toward the neuronal mechanism of hunting behavior.

## 2. Results

### 2.1. LH CaMKII*α*^+^ Neurons Encode Novelty-Seeking Behavior during Novel Object Exploration

**Figure 1:**
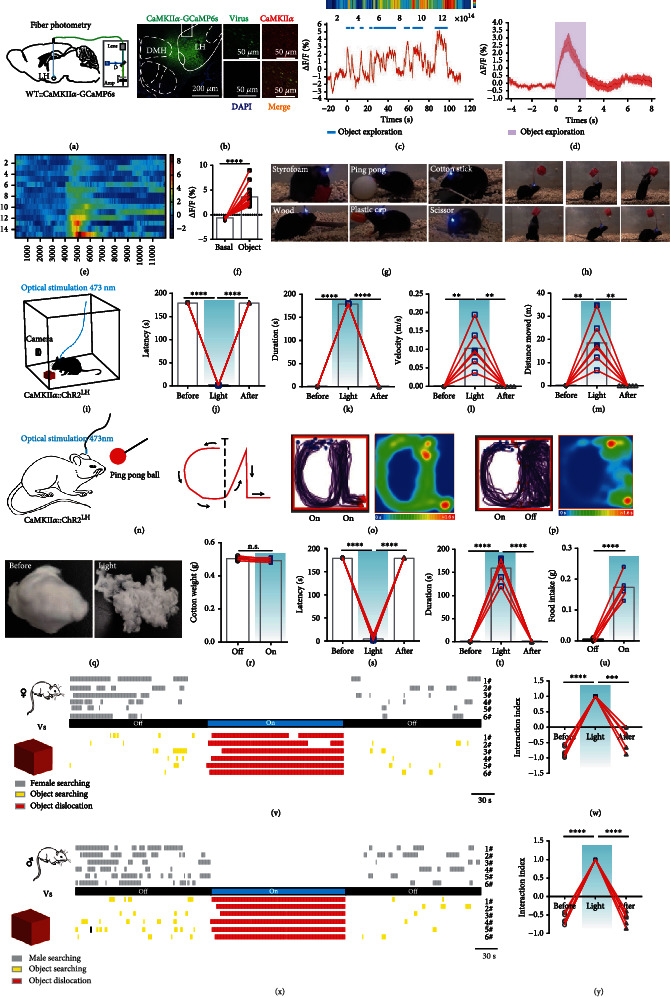
LH CaMKII*α*^+^ neurons encode novelty-seeking signals in object exploration. (a) Schematic image of fiber photometry. (b) Left, representative image of AAV-CaMKII*α*-GCaMP6s virus and optical cannula position, scale bar, 200 *μ*m; right, colocalization of the AAV-CaMKII*α*-GCaMP6s virus (green) and immunostaining of CaMKII*α* (red), scale bar, 50 *μ*m. (c) Representative single raw neuronal activity and heat map when mice interacted with novel object. (d) Mean calcium activities of 5 mice (each mouse was repeated for three times). The purple area indicated the exploration process, and the error bar indicated SEM of all trials. (e) Heat map of fluorescence of 5 mice. (f) Quantification of fluorescence changes (*n* = 5; paired *t*-test, ^∗∗∗∗^*P* < 0.0001). (g) CaMKII*α*::ChR2^LH^ mice showed active interaction with different objects. (h) Mouse was leaping and reaching out to a hanging object. (i) Schematic of optogenetics and video record. (j–m) Effect of optical stimulation of CaMKII*α*^LH^ neurons on object exploration behavior: latency to initiate retrieve an object (j), duration of object dislocation and biting (k), average velocity of object dislocation (l), and distance moved when retrieving an object (m) (*n* = 6, one-way ANOVA followed by Dunnett's post hoc test, ^∗∗∗∗^*P* < 0.0001, ^∗∗^*P* < 0.01). (n–p) Effects on chasing a moving ball: schematic illustration of chasing experiment (n); cumulative traces and mean heat map with constant light delivery (o); and cumulative routes and mean heat map, the light was withdrawn after finishing contour “C” (p). (q–t) Effect of biting a cotton ball: representative cotton ball images (q), cotton weight (r), latency of biting (s), and duration of biting (t) (*n* = 6, one-way ANOVA followed by Dunnett's post hoc test, ^∗∗∗∗^*P* < 0.0001). (u) Food pellet consumption in 3 min (*n* = 6, paired *t*-test, ^∗∗∗∗^*P* < 0.0001). (v) Behavioral raster plots illustrated synchronous interaction with a Styrofoam cube and a female mouse. (w) The interaction index (*n* = 6; one-way ANOVA followed by Dunnett's post hoc test, ^∗∗∗∗^*P* < 0.0001, ^∗∗∗^*P* < 0.001). (x) Behavioral raster plots with a Styrofoam cube and a younger male mouse. (y) Interaction index (*n* = 6, one-way ANOVA followed by Dunnett's post hoc test, ^∗∗∗∗^*P* < 0.0001).

Brain neuronal activities accompanied by rapid changes in intracellular Ca^2+^ [14] modulate how we perceive and react to the stimuli of the surrounding environment [15–17]. GCaMP is a family of ultrasensitive Ca^2+^ sensor that offers a powerful strategy to monitor the real-time concentration of Ca^2+^ in the cytoplasm and reflects the neuronal activity [18]. Firstly, we monitored the activities of LH CaMKII*α*^+^ neurons when mice explored new stimuli which is the first procedure of hunting (Figure 1(a)). We targeted LH CaMKII*α*^+^ neurons by unilaterally stereotaxic infusion of AAV-CaMKII*α*-GCaMP6s virus of WT mice (Figure 1(b)) and implanted optical fibers above the LH. To ensure the virus specificity, we immunolabeled CaMKII*α* (Figure 1(b)); out of 857 GCaMP6s infected neurons from 2 mice in LH, 844 cells were CaMKII*α*^+^, and the colocalized proportion was 98.48%, indicating that the virus was credible. Upon introduction of a novel object in a familiar circumstance, mice constantly explored it out of instinct; we observed that once mouse contacted the novel object with their nose, the fluorescent signal increased sharply; what is more, when mice retrieved the object with their mouth, the fluorescence of GCaMP maintained at high level continuously and fell down immediately when they put it down (Figure 1(c) and Video S1), and 5 experimental mice exhibited similar neuronal trends toward novel objects (Figures 1(d) and 1(e)). A significant increase in the Ca^2+^ signal ratio (the ratio of measurements taken 2 s before and 2 s after the nose contacting the object) can be observed (Figure 1(f)). The results above demonstrated that LH CaMKII*α*^+^ neurons are involved in novelty-seeking and object retrieval behaviors.

### 2.2. Activation of LH CaMKII*α*^+^ Neurons Promotes Hunting-Like and Feeding Behaviors

To further investigate the causal function of LH CaMKII*α*^+^ neurons, we injected AAV-CaMKII*α*-ChR2-EYFP virus into the LH of male WT mice and implanted optical fibers to manipulate cellular activity of LH CaMKII*α*^+^ neurons by optogenetics. By post hoc test, we confirmed that our targeting was limited to the LH and optical stimulation was efficient. In a transparent arena with the red Styrofoam cube (Figure 1(i)), 473 nm blue light can immediately evoke mice to explore the whole arena; once they encountered the red Styrofoam cube, they showed great interest in the object and touched the object by their nose and two forepaws, and then, they retrieved the object with their mouth and quickly traveled around the whole arena and sometimes they would stop to bite the cube (Video S2). Next, different sizes and material objects including plastic ball, wood, cotton stick, plastic cap, or scissor were used to repeat the experiment (Figure 1(g)). Mice showed different styles of interactions with different objects. They used their mouths to carry short wooden sticks. However, if the size was too big, like the ping-pong ball, the mice would hug the ping-pong ball with two forepaws and attempt to bite it (Video S2). The exploration latency was largely shortened (Figure 1(j)) and the object dislocation duration was greatly lengthened (Figure 1(k)), and the mice with Styrofoam cubes in their mouths moved observably faster and further (Figures 1(l) and 1(m)) when LH CaMKII*α*^+^ neurons were activated. To determine whether tactile sense was necessary for the object exploration behavior, the object was suspended in the air, in which situation, mice cannot sense the object with their whiskers and had no way to acquire the object without jumping up. Once we activated the LH CaMKII*α*^+^ neurons, mice leaped up to reach the hanging object, bite it tightly, and then fell off (Figure 1(h)); this stated that the LH CaMKII*α*^+^ neuron-mediated object exploration behavior was independent of tactile sense but relied on the visual system. In summary, these above results illustrated that mice searched the environment and retrieved nonsocial objects once LH CaMKII*α*^+^ neurons were activated. Many species naturally show object-carrying behavior, which by some species may be viewed as a basic, adaptive response that under specific circumstances serves the purposes of specific motivational states. This is apparent in situations of food hoarding, nest building, pup retrieval, or having no obvious utility for the animal at all [19]; we then test whether LH CaMKII*α*^+^ neurons mediate hunting.

Since predatory hunting is a sequential action including exploration, chasing, biting, grabbing, and retrieval, we progressively tested the behaviors described above. Fear is a fundamental emotion, mice exhibit strong fear responses when exposed to fast-moving objects, probably because these resemble visual features of a natural predator indicated threats and will elicit defensive behavioral responses like actively fight, flight, or positively freezing behaviors, which enable the animals to avoid or reduce harm and survive [20, 21]. To test whether LH CaMKII*α*^+^ neuron activation will turn mice from frightened emotion to predatory attack, we tied a ping-pong ball on a stick and manually approached mice along the designed route “CL” (Figure 1(n)) which is the acronym of “Chen Lab.” Mice were fearful of the object and stayed away from the object when stimulation was off. On the contrary, mice chased the ping-pong ball closely and tried to bite it when stimulation was on; ping-pong ball was moved along “CL” and the trace of mice also shaped striking like “CL” under constant light stimulation (Figure 1(o)); however, mice stopped chasing immediately and moved randomly (Figure 1(p)) when we removed the light after they finished the “C” portion (Video S2). Next, we explored whether mice would bite the object when LH CaMKII*α*^+^ neurons were activated; we put a 0.5 g cotton ball into the arena. Mice would voraciously bite the cotton ball when LH CaMKII*α*^+^ neurons were activated. After optical stimulation for 3 min, the cotton ball was torn into pieces (Figure 1(q)). The cotton weight did not decrease (Figure 1(r)), and the biting latency was significantly shortened (Figure 1(s)), while the biting duration was remarkably lengthened (Figure 1(t)) upon optical stimulation, stating that CaMKII*α*::ChR2^LH^ activation induced a strong desire to bite object. In consideration of LH is import for feeding, we further explored the effect of CaMKII*α*::ChR2^LH^ activation on feeding behavior. Well-fed mice bit food pellets voraciously and swallowed them once the LH CaMKII*α*^+^ neurons were activated (Video S3). There was a significant difference of consumed food weight between the stimulation off and stimulation on phase (Figure 1(u)), and laser activation also abolished natural preferences for esculent over nonesculent objects (Video S4). These results demonstrated that LH CaMKII*α*^+^ neuronal activation promoted chasing and nonselective feeding behaviors.

Animals constantly interact with their environment to identify useful information including food sources or to communicate with conspecifics to find mating [22–24]. To verify whether activating LH CaMKII*α*^+^ neurons make male mice prefer nonsocial object to social interaction, we performed competitive assays using a nonsocial object and an unacquainted female in estrus introduced and calculated the time mice spent on exploring object and conspecifics, respectively. During the light off stage, male mice spent most time on typical courtship behaviors (chasing, sniffing, and mounting) toward the female (Figure 1(v)). However, a remarkable increase of attention toward the object rather than the female was observed when LH CaMKII*α*^+^ neurons were activated (Figure 1(w)). Consequently, mice could discriminate social and nonsocial targets and generate a strong drive to shift their attention from social interaction toward nonsocial objects when LH CaMKII*α*^+^ neurons are activated. Together, the above results demonstrated that activating LH CaMKII*α*^+^ neurons elicit more of a tendency for nonsocial object exploration behavior rather than social interaction.

The general function of aggression refers to two categories: social aggression and predation [25, 26]. Social aggression refers to a hostile behavior toward conspecifics to protect resources such as diet, reproductive partners, territory, or social rank [27]. Conversely, predation targets different species to kill and eat them to replenish energy. Social aggression is frequently observed in laboratory male mice using a resident-intruder assay, and the original resident often initiates aggressive actions [28]. To distinguish whether the aggressive behavior was derived from predatory hunting or social aggression, we introduced a strong and aggressive male mouse and a novel object into the arena with the experimental mice. We observed mice interacted with both the male mouse and object but spent more time with the strange male in the light off phase. However, mice concentrated on the object immediately when the light was delivered and retrieved the object rather than confronting the male mouse; we failed to observe any occurrence of attack during the stimulation stage. After removal of the optical stimulation, mice put down the object immediately (Figures 1(x) and 1(y)). It illuminates that LH CaMKII*α*^+^ neurons do not mediate social aggression behavior but predatory attacks.

### 2.3. LH CaMKII*α*^+^ Neurons Bidirectionally Modulate Predatory Attack and Eating in Hunting Behavior

**Figure 2:**
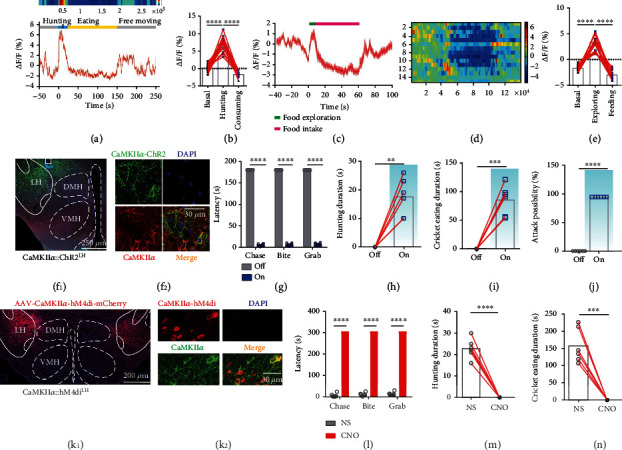
LH CaMKII*α*^+^ neurons bidirectionally modulate predatory attack and eating in hunting behavior. (a) Representative raw trace and heat map during predation: hunting process (blue), cricket consumption process (yellow), and free movement (gray). (b) Average GCaMP signal aligned to the start of attack and prey consumption (each of five mice was repeated for three times, one-way ANOVA followed by Dunnett's post hoc test, ^∗∗∗∗^*P* < 0.0001). (c) Mean calcium signal activities during feeding behavior (each of five mice was repeated for three times), food exploration (green), and feeding (red) were shown, and the error bar indicated SEM. (d) Heat map of fluorescence alteration (each of five mice was repeated for three times). (e) Mean GCaMP signal of all mice aligned to food retrieval (each of five mice was repeated for three times, one-way ANOVA followed by Dunnett's post hoc test, ^∗∗∗∗^*P* < 0.0001). (f–j) Effects of activation LH CaMKII*α*^+^ neurons on hunting. (f_1_) Representative image of CaMKII*α*::ChR2^LH^ expression and optical cannula position and Fos immunolabeling, scale bar, 200 *μ*m. (f_2_) Colocalization of the AAV-CaMKII*α*-ChR2-EYFP virus (green) and immunostaining of CaMKII*α* (red), scale bar, 30 *μ*m. Latency of hunting a cricket (g), duration of hunting (h), duration of consuming (i), and attack possibility of 5 crickets (j) (*n* = 6, paired *t*-test, ^∗∗∗∗^*P* < 0.0001, ^∗∗∗^*P* < 0.001, ^∗∗^*P* < 0.01). (k–n) Effect of chemogenetic inhibition of CaMKII*α*^LH^ neurons on hunting behavior. (k_1_) Representative image virus expression, scale bar, 200 *μ*m. (k_2_) Colocalization of the hM4di-mCherryexpression and immunostaining of CaMKII*α* (green), scale bar, 30 *μ*m. Latency of a sequential hunting (l), duration of hunting (m), and duration of cricket consumption (n) (*n* = 6, paired *t*-test, ^∗∗∗∗^*P* < 0.0001, ^∗∗∗^*P* < 0.001).

Since LH CaMKII*α*^+^ neuron activation induces mice to search, chase, bite, and retrieve objects which behaves like hunting, we examine whether LH CaMKII*α*^+^ neurons are involved in hunting. To evaluate the performance of LH CaMKII*α*^+^ neurons during active hunting, we monitored Ca^2+^ changes during starvation-induced cricket hunting paradigm. When lively crickets were introduced into an arena where food-deprived mice existed, alignment of the GCaMP signals with video-recorded behavior revealed that the delivery of bites aimed at a cricket was accompanied by a sharp increase in GCaMP fluorescence, which was maintained at a high level throughout the whole hunting action. Nevertheless, neuronal activity declined during the following consumption (Figures 2(a) and 2(b) and Video S1). We did not detect any obvious changes in fluorescence signals (Figure 2(a)) in LH CaMKII*α*^+^ neurons during free movement, indicating that the GCaMP signals genuinely indicated neuronal activity and did not simply reflect locomotion. We further investigated the neuronal activity when food-deprived mice were given a normal food pellet. Mice first interacted with the food pellet to make sure edibility. A sharp transient fluorescence signal increase was observed when they interacted with the food, which showed the same trends as Styrofoam exploration previously. Then, the fluorescence signal sharply fell when they consumed the food and the neuronal activity returned to baseline when the mice finished eating (Figures 2(c)–2(e)). We observed that Ca^2+^ fluorescence of LH CaMKII*α*^+^ neurons decreased when consumed food pellet and crickets; however, activating LH CaMKII*α*^+^ neurons promoted hunting and feeding behaviors; we assumed that LH CaMKII*α*^+^ neurons may encode appetite. Food-deprived mice were hungry and originated an appetite for food, so LH CaMKII*α*^+^ neurons were highly active when mice did not get food. However, once mice got food supply, the appetite was inhibited immediately. All those results demonstrate that LH CaMKII*α*^+^ neurons are involved in hunting and feeding behaviors.

Then, we expressed AAV-CaMKII*α*-ChR2-EYFP within the LH (Figure 2(f_1_)); out of 591 ChR2-EYFP expressed neurons in the LH, 578 neurons were immunostaining positive for CaMKII*α*, and the colocalization percentage was 97.80% (Figure 2(f_2_)), proving the specificity of AAV-CaMKII*α*-ChR2-EYFP virus. Then, we examined the effect of LH CaMKII*α*^+^ neuronal activation on the inclination and ability to hunt. Since animals naturally avoid rapidly approaching objects which they perceive as threats, we first introduced a plastic electrical artificial prey. This electrical prey was inedible but it moved fast and can avoid obstacles to mimic the natural prey. Mice evaded it in the light off phase. On the contrary, CaMKII*α*::ChR2^LH^ neuron activation induced mice to chase, bite, grab, and restrain the moving artificial prey instantly (Video S5). Therefore, optical activation of LH CaMKII*α*^+^ neurons on artificial prey imitated the typical action observed during insect hunting, reminiscent of the natural hunting behavior [29]. We replaced the artificial prey into real crickets, results showed that the optogenetic stimulation of the CaMKII*α*::ChR2^LH^ of cricket-naïve and well-fed mice induced a robust hunting attack, the prey captured was assisted by pinning the prey to the substrate with the forepaws, and oral grasping the prey with the forepaws either simultaneously or shortly after the killing bite was administered toward the prey's head followed by voracious consuming of the dead body (Video S5). However, once stimulation was withdrawn, the animal would dump the cricket immediately and ignore it completely. Optical stimulation reduced the hunting latency (Figure 2(g)) and lengthened hunting duration (Figure 2(h)) and cricket eating duration (Figure 2(i)) relative to the light off phase. Furthermore, mice would attack and consume all of the given 5 crickets when optically stimulated (Figure 2(j)). These results demonstrate that activation of LH CaMKII*α*^+^ neurons drives mice to attack against artificial or live crickets and them.

To explore if the endogenous activity of LH CaMKII*α*^+^ neurons is necessary for hunting, we used a chemogenetic strategy [30] to inhibit the neuronal firing. LH was bilaterally injected with AAV-CaMKII*α*-hM4Di-mCherry virus (Figure 2(k_1_)); out of 495 hM4di-mCherry expressed neurons in the LH, 482 neurons were immunostaining positive for CaMKII*α*, and the colocalization percentage was 97.37% (Figure 2(k_2_)), proving that the AAV-CaMKII*α*-hM4di-mCherry virus was credible. Mice were intraperitoneally injected with normal saline and clozapine N-oxide (CNO), fasting mice lost interest in crickets of the CNO group compared with those which have been injected with normal saline (Figures 2(l)–2(n)). These results indicate that LH CaMKII*α*^+^ neurons are sufficient and necessary to encode predatory eating in hunting behaviors.

### 2.4. CaMKII*α*^LH-vPAG^ Projection Encodes Hunting Behavior

**Figure 3:**
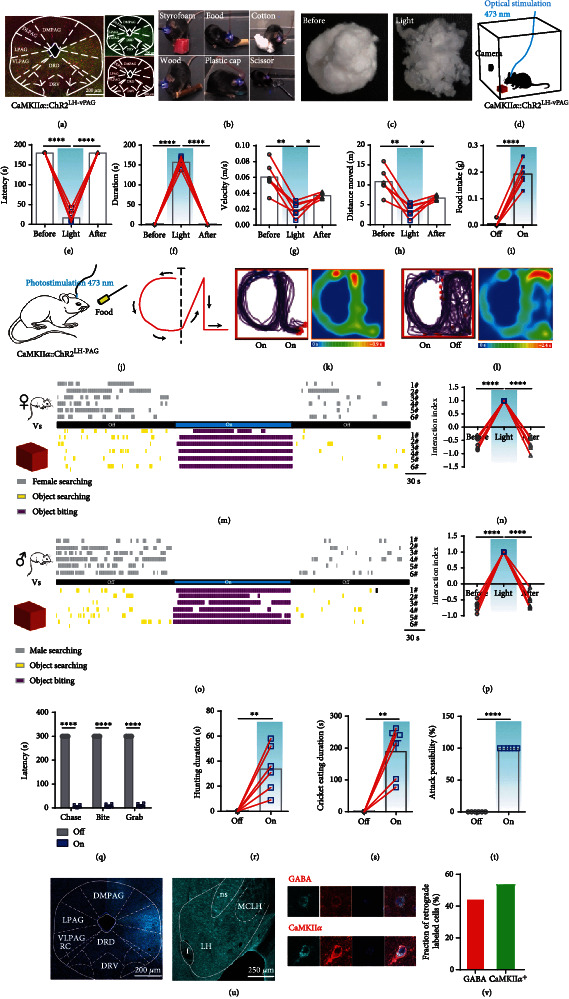
CaMKII*α*^LH-vPAG^ circuit mediates hunting behavior. (a) Representative image of LH-vPAG axon terminals (green) of AAV-CaMKII*α*-hChR2-EYFP infected neurons and Fos immunolabeling (red) after 30 min of stimulation of the projection, scale bar, 200 *μ*m. (b) When stimulated CaMKII*α*::ChR2^LH-vPAG^, mice biting rather than dislocating objects. (c) Representative small cotton ball images before and after stimulation. (d–h) Effect of activation of the CaMKII*α*^LH-vPAG^ circuit on object biting behavior. (d) Latency (e) and duration (f) of biting, average velocity (g), and distance moved (h) in the open field test (*n* = 6, one-way ANOVA followed by Dunnett's post hoc test, ^∗∗∗∗^*P* < 0.0001, ^∗∗^*P* < 0.01, ^∗^*P* < 0.05). (i) Effect of activation CaMKII*α*^LH-vPAG^ on food intake (*n* = 6, paired *t*-test, ^∗∗∗∗^*P* < 0.0001). (j–l) Effect of CaMKII*α*^LH-vPAG^ mice on chasing caloric food. Schematic showing that mice chased caloric food along the guiding route (j); merged traces and mean heat map under constant light delivery (k); and merged routes of the mouse following pathways and mean heat map, the light was withdrawn immediately after contour “C” finished (l). (m) Behavioral raster plots when CaMKII*α*::ChR2^LH-vPAG^ was activated. Behaviors toward females and objects were, respectively, illustrated. (n) The interaction index toward female and object (*n* = 6; one-way ANOVA, Dunn's multiple comparisons test, ^∗∗∗∗^*P* < 0.0001). (o) Behavioral raster plots of male mice with a Styrofoam cube and a younger male mouse. (p) The interaction index of the male mouse and object (*n* = 6; one-way ANOVA, Dunn's multiple comparisons test, ^∗∗∗∗^*P* < 0.0001). (q–t) Effect of CaMKII*α*::ChR2^LH-vPAG^ activation on hunting crickets of ad libitum feeding mice. Latency of hunting (q), hunting duration (r), cricket consumption duration (s), and attack possibility of 5 crickets (t) (*n* = 6, paired *t*-test, ^∗∗∗∗^*P* < 0.0001, ^∗∗^*P* < 0.01). (u) Left, representative image of CT-B 647 in the vPAG, scale bar, 200 *μ*m; middle, representative image of retrograde soma in LH, scale bar, 250 *μ*m; right, colocalization of CT-B 647 retrograde soma and immunolabeled of GABA and CaMKII*α*. (v) Percentage of retrograde labeled cells of GABAergic and CaMKII*α*^+^ neurons.

We next sought to identify the output pathway of LH CaMKII*α*^+^ neurons. Previous research revealed that PAG is a key region for executing predatory hunting. As it is also one of the output regions of the LH, we speculated that the PAG is the downstream target. The immunohistochemical results indicated that vPAG consisting of lPAG and vlPAG is a major subregion synaptic target of LH CaMKII*α*^+^ neurons, and after the activation of LH CaMKII*α*^+^ neurons, Fos protein was observed in the vPAG (Figure 3(a)). Different from the LH CaMKII*α*^+^ soma stimulation, CaMKII*α*^LH-vPAG^ activated mice just bit the object and remained still but did not dislocate it (Figure 3(b)). The cotton ball was torn into pieces after the optical stimulation (Figure 3(c)), suggesting that CaMKII*α*^LH-vPAG^ projection activation induced an excessive level of oral movement but not for the whole body. We quantified the biting behavior using the paradigm as described before (Figure 3(d)). The biting latency was significantly decreased (Figure 3(e)) and the duration was markedly lengthened (Figure 3(f)), illuminating that light-evoked intense biting behavior. However, the average velocity (Figure 3(g)) and distance moved (Figure 3(h)) decreased significantly after mice reached the cotton because the mice were immersed in biting. To investigate whether this behavior was feeding or just simple chew, an edible food pellet was introduced into the box and the amount of food consumed was measured; the result indicated that CaMKII*α*^LH-vPAG^ activation evoked voracious feeding behavior (Figure 3(i)). Further, to explore whether CaMKII*α*^LH-vPAG^ activation would induce chasing-like behavior, we first used the ping-pong ball to navigate the mice as before. Interestingly, mice rarely followed the ping-pong ball (Video S6), suggesting a lack of desire for the inedible object. Therefore, we replaced the ping-pong ball with caloric food (Figure 3(j)). Upon light delivery, mice were eager for food, as they chased the fast-moving food along with the contour “C” and “L” and moved randomly when stimulation was off (Figures 3(k) and 3(l) and Video S6). CaMKII*α*^LH-vPAG^ activation also mediated the preference to an object rather than to a female (Figures 3(m) and 3(n)), and we did not observe any occurrence of social aggression compared with CaMKII*α*^LH-vPAG^ activation (Figures 3(o) and 3(p)). These results illustrate that CaMKII*α*^LH-vPAG^ activation selectively evokes desire for caloric food.

We further investigated the effects of CaMKII*α*^LH-vPAG^ projection on live crickets. Optical stimulation evoked mice to pounce on the cricket and attack using the mouth and forepaws savagely (Figure 3(q)). After restraining the cricket, mice ate the cricket rapidly (Figures 3(r) and 3(s)). All crickets were hunted and rapidly consumed in 5 min (Figure 3(t)). This indicates that CaMKII*α*^LH-vPAG^ projection encodes appetite-driven hunting and that mice can distinguish inedible objects from edible objects.

A recent study revealed that a GABAergic circuit from the LH to the PAG promotes predatory hunting, but mice will not consume the dead crickets [11]. Meanwhile, previous study also uncovered that separate LH GABAergic neurons selectively encode aspects of appetitive or consummatory behaviors [31]. To figure out what kind of neuronal type of LH CaMKII*α*^+^ neurons belong to, we crossed *vGaT-cre* mice with report mice *Ai47* to mark the GABAergic neurons with enhanced green fluorescence and then immunolabeled CaMKII*α*^+^ neurons to calculate colocalization percentage. We found that the LH CaMKII*α*^+^ neurons scarcely overlapped with GABAergic neurons (Figure S1(a)). We applied the same strategy to verify the colocalized proportion of CaMKII*α*^+^ and vGluT2^+^, upon which we found that out of 426 vGluT2^+^ neurons in the targeted LH, 412 cells were positive for CaMKII*α*, and the colocalized proportion was 96.71%; out of 645 CaMKII*α*^+^ neurons in the LH, 412 cells were positive for vGluT2, and the colocalized proportion was 63.87% (Figure S1(b)), suggesting that LH CaMKII*α*^+^ are partially vGluT2^+^ glutamatergic neurons but are not GABAergic neurons. Furthermore, we made a retrograde tracing of vPAG and immunostaining GABA and CaMKII*α* in LH separately (Figure 3(u)). Out of 91 vPAG-projecting LH neurons, 49 neurons were colocalized with CaMKII*α*, and 53.85% were putatively CaMKII*α*^+^; and out of 151 vPAG-projecting LH neurons, 67 neurons were colocalized with GABA, and the colocalization percentage was 44.37% (Figure 3(v)); these results illustrate that vPAG receives both GABAergic and CaMKII*α* projection from LH.

However, in previous study, photoactivation of LH vGluT2^+^ neurons suppressed feeding [32]. We next verify whether LH CaMKII*α*^+^vGluT2^−^ neurons are responsible for promotion of hunting and feeding behavior. AAV-CaMKII*α*-DO-GCaMP6s virus was injected into LH of *vGluT2-cre* mice and optical cannulas were implanted above it; thus, the virus can infect LH CaMKII*α*^+^ vGluT2^−^ neurons (Figure S1(c)). Then, mice were food-deprived for 12 h and were introduced with crickets, and mice predated crickets fiercely out of innate starvation; we observed that the fluorescence signal increased immediately once mice chased crickets and maintained a high level during biting and grabbing (Figure S1(d-e)). Meanwhile, we injected AAV-DIO-casepase3 virus into the LH of *vGluT2-cre* mice to kill vGluT2^+^ neurons and then infected the LH with AAV-CaMKII*α*-ChR2-EYFP; the representative images showed that AAV-CaMKII*α*-ChR2-EYFP virus had rich expression even though all LH vGluT2^+^ neurons were killed (Figure S1(f-g)). We surprisingly found that well-fed mice, whose LH vGluT2^+^ neurons were killed, exhibited spontaneous hunting behavior toward crickets without optical activation, and optically activating LH vGluT2^−^ CaMKII*α*^+^ neurons induced faster and more fierce hunting behavior (Figure S1(k-n)) and food pellet consumption (Figure S1(o)). The results above proved that LH CaMKII*α*^+^ vGluT2^−^ neurons are indeed responsible for the promotion of hunting and feeding behavior.

### 2.5. LH CaMKII*α*^+^ Neurons Receive Monosynaptic Excitatory Inputs from MPOA

**Figure 4:**
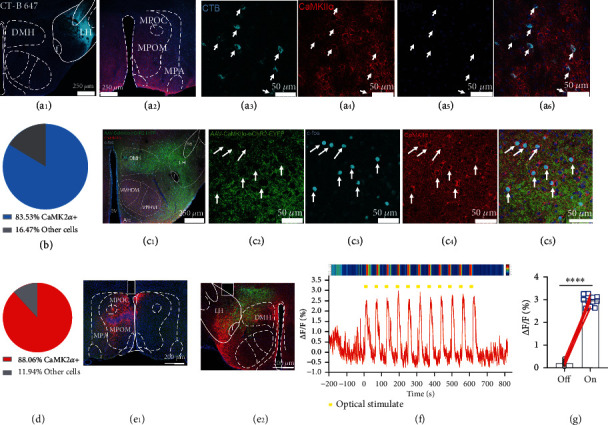
The LH CaMKII*α*^+^ neurons are directly modulated by MPOA CaMKII*α*^+^ neurons.(a_1_–a_6_) Retrograde tracing of LH. Representative image of CT-B 647 in LH, scale bar, 250 *μ*m (a_1_); representative image of retrograde soma in MPOA, scale bar, 250 *μ*m (a_2_); the enlarged view of CT-B tracing neuronal soma in MPOA (a_3_); immunostaining of CaMKII*α* (a_4_); DAPI (a_5_); and colocalization (a_6_). (b) Percentage of CaMKII*α*^+^ neurons accounts for all CT-B tracing neurons. (c_1_–c_5_) MPOA-LH function connection identification. Representative image of CaMKII*α*^MPOA-LH^ projection (green) and immunostaining of Fos (cyan) and CaMKII*α* (red) after optical stimulation, scale bar, 250 *μ*m (c_1_); the enlarged images of ChR2-EYFP expression (c_2_); immunostaining of c-Fos (c_3_); CaMKII*α* (c_4_); and their colocalization (c_5_), scale bar, 50 *μ*m. (d) The percentage of CaMKII*α*^+^ neurons accounted for all immunolabeled Fos^+^ neurons. (e_1_) Representative image of CaMKII*α*::ChrimsonR^MPOA^, scale bar, 200 *μ*m. (e_2_) Representative image of CaMKII*α*::GCaMP6s^LH^ (green), scale bar, 200 *μ*m. Red color indicates ChrimsonR-mCherry projection. (f) Representative raw trace and heat map of the fluorescence signal of LH CaMKII*α*^+^ neurons corresponding to MPOA CaMKII*α*^+^ neuron activation; yellow bars indicate each optical stimulation. (g) Average GCaMP signal of all trials aligned to the optical stimulation (paired *t*-test, ^∗∗∗∗^*P* < 0.0001).

Then, to dissect the upstream neuronal circuits of LH, we performed a cholera toxin subunit B (CT-B) retrograde tracing strategy to search upstream nucleus where LH receives predation-related information from. CT-B conjugating with fluorophore can be absorbed by axon terminal and retrograde tracing to neuronal soma. CT-B conjugated with Alex 647 was restricted expressed in the LH (Figure 4(a_1_)), and whole brain upstream nuclei were mapped; we noticed that LH received dense projection from the MPOA (Figures 4(a_2_) and 4(a_3_)), which is anterior part of the hypothalamus, critically regulating social behaviors and reward, including maternal behavior [33], sexual behavior [34], thermoregulation [35], and novel object exploration [13]. We immunolabeled CaMKII*α* (Figure 4(a_4_)) and calculated the percentage of colocalization (Figure 4(a_5_)), 340 LH-projecting MPOA neurons were analyzed and 284 neurons were found colocalized with CaMKII*α*, and the percentage was 83.53% (Figure 4(b)), which illustrates that LH-projecting MPOA neurons are mainly CaMKII*α*^+^. Further, we made anterograde tracing by injecting AAV-CaMKII*α*-hChR2-EYFP virus into the MPOA of adult male C57BL/6J mice and optical activated the soma for half an hour and immunolabeled whole brain immediate early gene product Fos protein. There were varying degrees of immunolabeled Fos in downstream nucleus target of MPOA CaMKII*α*^+^ neurons (Figure S2(a)). It is worth noting that there were abundant axons (Figures 4(c_1_) and 4(c_2_)) and Fos protein (Figure 4(c_3_)) in the LH and previously reported nucleus PAG (Figure S2a), suggesting the importance of these two targets. Next, we explored the type of neurons in LH that the CaMKII*α*^MPOA-LH^ circuit acted on. After optical stimulation of MPOA CaMKII*α*^+^ neurons for 30 min, Fos and CaMKII*α* (Figure 4(c_4_)) immunolabeling illustrated that 88.06% of the 243 Fos^+^ cells in LH were CaMKII*α*^+^ neurons (Figure 4(d)). To further investigate the functional connection of MPOA CaMKII*α*^+^ neurons and LH CaMKII*α*^+^ neurons, we performed fiber photometry strategy. *CaMKIIα-cre* mice were injected with AAV-hSyn-DIO-ChrimsonR-mCherry [36] in MPOA to optically stimulate MPOA CaMKII*α*^+^ neurons (Figure 4(e_1_)); meanwhile, LH CaMKII*α*^+^ neurons were infected with AAV-EF1*α*-DIO-GCaMP6s (Figure 4(e_2_)) to monitor real-time neuronal activity. The results showed that optogenetic activation of MPOA CaMKII*α*^+^ neurons directly enhanced Ca^2+^ concentration in LH CaMKII*α*^+^ neurons (Figures 4(f) and 4(g)), demonstrating that MPOA CaMKII*α*^+^ neurons directly excite LH CaMKII*α*^+^ neurons.

### 2.6. MPOA CaMKII*α*^+^ Neuron Activation Promotes Object Exploration and Nonappetitive Hunting Behavior

Since MPOA and LH are structurally connected, we assume whether MPOA CaMKII*α*^+^ neurons encode novel-seeking behavior. To quantify the physiological activity of the MPOA CaMKII*α*^+^ neurons during object exploration, we monitored Ca^2+^ activity of MPOA CaMKII*α*^+^ neurons (Figure S3(a)). 96.71% of the 243 neurons were colocalized with CaMKII*α* immunostaining (Figure S3(b)), verifying the virus specificity. We noticed a significant increase of fluorescence signals in the MPOA CaMKII*α*^+^ neurons when mice explored the novel object (Figure S3(c-f)).

Optogenetic stimulation of MPOA CaMKII*α*^+^ neurons showed object exploration behaviors (Figure S3(g-j)), precisely chased moving objects along “C” and “L” (Figure S3(k-m)), bit cotton balls, and shifted attention toward the object from the female (Figure S3(s-t)). However, different from LH CaMKII*α*^+^ neuron activation, MPOA CaMKII*α*^+^ neuron activation induced food pellet dislocation rather than feeding behavior (Figure S3(r) and Video S3). In conclusion, these results suggest that MPOA CaMKII*α* neurons are essential for mice to engage with objects including biting, chasing, and retrieving. However, as opposed to LH CaMKII*α*^+^ neurons, MPOA CaMKII*α*^+^ neurons do not encode forced feeding behavior.

Further, we investigate whether MPOA CaMKII*α*^+^ neurons participate in hunting behaviors. Neuronal activity of MPOA CaMKII*α*^+^ neurons increased prominently when mice predated a cricket, then decreased when mice consumed the body, and returned to baseline when mice finished consumption (Figure S4(a-c)). Optical activation of MPOA CaMKII*α*^+^ neurons turned mice to pursue, bite, and capture artificial prey. Furthermore, optogenetically activated MPOA CaMKII*α*^+^ neurons enhanced mice to move efficiently toward the moving prey, induced a robust hunting attack with a sequence of pursuit, the release of biting attacks, restrain, and retrieval. After stimulation, the mice would dump the cricket immediately and ignore it completely and stand poised over it. Interestingly, mice would not consume the dead crickets which was killed by them and leave the body on the floor (Figure S4(d-i)). Next, a cell type-specific silencing approach with archaerhodopsin (Arch), a light-driven outward proton pump that can mediate powerful silencing of neural activity, was used [37]. AAV-CaMKII*α*-ArchT-EGFP was expressed in the MPOA of WT mice (Figure S4(j1)); out of 552 ArchT-EGFP expressed neurons in MPOA, 547 neurons were immunostaining positive for CaMKII*α*, and the colocalization percentage was 99.09% (Figure S4(j2)), proving that AAV-CaMKII*α*-ArchT-EGFP virus was specific. Optogenetic inhibition of MPOA CaMKII*α*^+^ neurons could abolish the action of predation driven by instinctive appetite (Figure S4(k-m) and video S7) but would not affect locomotion distance (Figure S4(n)) and velocity (Figure S4(o)). Next, we employed another loss of function method by electrical lesion of the MPOA, an electrode was implanted into MPOA, and direct current was given for lesion (Figure S4(p)). After food deprivation for 12 h, MPOA-lesioned mice did not attempt to chase and hunt crickets within 5 min, unlike the sham group mice (Figure S4(q-s)), while MPOA lesion did not affect normal food intake (Figure S4(t)). Thus, these above findings illuminate that the activity of MPOA CaMKII*α*^+^ neurons is sufficient and necessary for nonappetitive hunting behaviors.

### 2.7. The CaMKII*α*^MPOA-LH^ Pathway Mediates Object Exploration and Hunting

**Figure 5:**
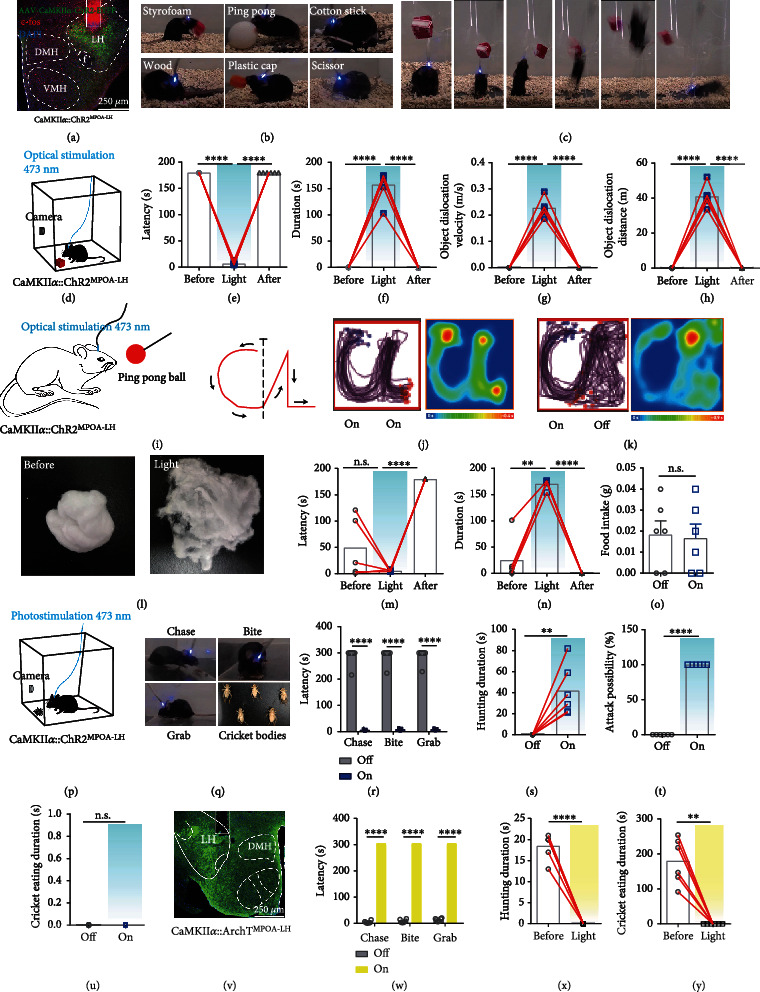
CaMKII*α*^MPOA-LH^ projection induces object exploration and hunting but not feeding behavior. (a) Representative image of MPOA-LH axons (green) and immunolabeled Fos protein (red), scale bar, 250 *μ*m. (b) CaMKII*α*::ChR2^MPOA–LH^ mice showed active interaction with different objects. (c) Mice showed a sequential posture of leaping and jumping up to reach an impending object. (d) Schematic of object dislocation behavior. (e–h) Effects of CaMKII*α*::ChR2^MPOA-LH^ activation on object exploration behavior: latency (e), duration (f), average object dislocation velocity (g), and object dislocation distance (h) (*n* = 6, one-way ANOVA followed by Dunnett's post hoc test, ^∗∗∗∗^*P* < 0.0001). (i–k) Effect of optical stimulation of MPOA-LH axon terminal upon chasing. Mice follow the moving ball along designed route (i); cumulative traces of the mouse trace and average heat map, the light was delivered constantly (j); cumulative traces of navigation pathways and mean heat map, the light was withdrawn immediately when finished “C” (k). (l–n) Effect of MPOA-LH projection activation on biting: representative image of cotton balls (l), latency (m), and duration of biting cotton (n) (*n* = 6, one-way ANOVA followed by Dunnett's post hoc test, ^∗∗^*P* < 0.01, ^∗∗∗∗^*P* < 0.0001). (o) Food intake in three min (paired *t*-test, *P* = 0.7926). (p) The experimental arena for hunting. (q–u) Effects of optical stimulation CaMKII*α*^MPOA-LH^ axon terminals on hunting behavior of well-fed mice: representative video snapshots of mice showed a sequential posture of hunting a cricket and the dead bodies (q), latency of each procedure when hunting (r), duration of hunting(s), attack possibility of 5 crickets (t), and cricket eating duration (u) (*n* = 6, paired *t*-test, ^∗∗∗∗^*P* < 0.0001, ^∗∗^*P* < 0.01). (v) Representative image of CaMKII*α*::ArchT^MPOA-LH^ axon terminals and optical fiber cannula position, scale bar, 200 *μ*m. (w and x) Effects of optical inhibition of the CaMKII*α*::ArchT^MPOA-LH^ axon terminal on hunting behavior of food-restricted mice: latency of each process (w) and duration of hunting a cricket (x) and cricket eating duration (y) (*n* = 6, paired *t*-test, ^∗∗∗∗^*P* < 0.0001, ^∗∗^*P* < 0.01).

Then, we focused on verifying the structural nature of the MPOA-LH circuit. To directly test whether the MPOA-LH circuit functionally mediates object exploration behavior. We infected MPOA with the AAV-CaMKII*α*-hChR2-EYFP virus and implanted optical fibers. Immunohistochemical labeling showed that activation of axon terminals strongly induced immunolabeled Fos in the LH, suggesting LH neuron activation (Figure 5(a)). Interestingly, light delivered to CaMKII*α*::ChR2^MPOA–LH^ axon terminal provoked object exploration behavior similar to the soma activation of MOPA CaMKII*α*^+^ neurons (Figure 5(b)). CaMKII*α*::ChR2^MPOA–LH^ activated mice targeted the object specifically and jumped up to reach hanging object (Figure 5(c)). Mice would also retrieve objects when CaMKII*α*::ChR2^MPOA–LH^ axons were activated in the test arena (Figures 5(d)–5(h)). Meanwhile, mice performed strikingly chasing (Figures 5(i)–5(k)) and biting (Figures 5(l)–5(n)) behavior. Activation of the CaMKII*α*^MPOA-LH^ projection mediated the preference to an object rather than a female (Figure S5(a-b)) but was not involved in social aggression behavior (Figure S5(c-d)). In conclusion, these data elucidated that CaMKII*α*^MPOA-LH^ projection is important for mice to engage with objects including exploring, biting, chasing, and retrieval.

To examine the functional effect of CaMKII*α*^MPOA-LH^ stimulation on hunting, we explored the effect of the projection on predating real prey. Optogenetic stimulation of the CaMKII*α*^MPOA-LH^ circuit of well-fed mice successfully induced a hunting attack toward a live cricket, where mice displayed pursuit, attack, capture, and retrieval behaviors (Figures 5(p)–5(t)) but did not consume the dead cricket (Figure 5(u)). We further found that mice would retrieve food pellets and moved rapidly in the arena but did not consume it (Figure 5(o)). To verify whether the MPOA-LH circuit was necessary for hunting, we optogenetically inhibited the CaMKII*α*^MPOA-LH^ circuit and found that mice demonstrated fierce predation toward crickets due to instinctive hunger. However, the predation behavior was extremely inhibited once the 589 nm light was delivered to inhibit this circuit (Figures 5(v)–5(y)). All of these results illustrate that the activation of the CaMKII*α*^MPOA-LH^ circuit triggers a strong motivation to prey on crickets but do not trigger the appetite to consume them.

### 2.8. MPOA-LH-vPAG Indirect Pathway Identification

**Figure 6:**
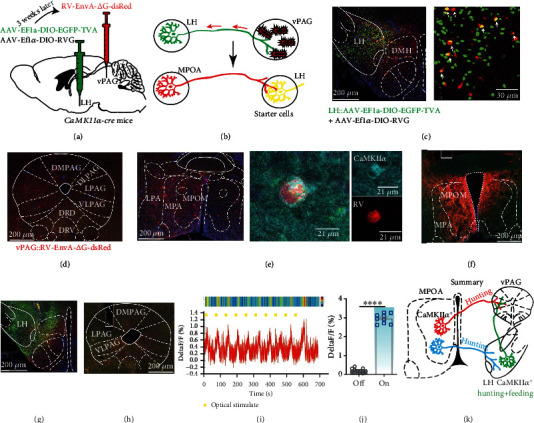
MPOA-LH-vPAG indirect pathway identification. (a) Schematic depiction of the virus injection protocol. (b) Schematic depiction of the rabies virus expression strategy. (c) Representative image of AAV-EF1a-DIO-His-EGFP-2*α*-TVA and AAV-EF1a-DIO-RVG and RV-EnvA-*Δ*G-dsRed in LH. The yellow cells indicate starter cells. (d) Representative image of RV-EnvA-*Δ*G-dsRed infected neural axons in vPAG, scale bar, 200 *μ*m. (e) Left, representative image of retrograde traced cells from LH in MPOA, scale bar, 200 *μ*m; middle, colocalization of RV-infected cells and immunolabeled cells of CaMKII*α*, scale bar, 21 *μ*m. (f) Representative image of AAV-hSyn-DIO-ChrimsonR-mCherry in MPOA of *CaMKIIα-cre* mice, scale bar, 200 *μ*m. (g) Representative image of AAV-Ef1*α*-DIO-axon-GCaMP6s expression in LH, scale bar, 200 *μ*m. (h) Representative image of PAG, scale bar, 200 *μ*m. (i) Representative raw trace and heat map of the fluorescence signal of LH projecting axons in vPAG corresponding to optogenetic activation of MPOA CaMKII*α*^+^ neurons. The yellow bars indicate each optical stimulation. (j) Average GCaMP signal of all trials aligned to the optical stimulation (paired *t*-test, ^∗∗∗∗^*P* < 0.0001). (k) Schematic summary of this research. MPOA CaMKII*α*^+^ neurons send indirect projections to the vPAG via LH CaMKII*α*^+^ neurons, which encodes novelty-seeking signal and encodes object exploration and appetite-driven hunting behaviors.

Further, to identify the indirect connection of MPOA-LH-vPAG pathway, the mixed helper virus AAV-Ef1*α*-DIO-EGFP-TVA and AAV-Ef1*α*-DIO-RVG were injected into LH of *CaMKIIα-cre* mice, the virus would infect the soma and axon, three weeks later, the retrograde virus RV-EnvA-*Δ*G-dsRed was injected into vPAG (Figure 6(a)), the RV-EnvA-*Δ*G-dsRed will be absorbed by the LH-projecting axons and retrograde tracing to the soma in LH, and neurons infected with AAV-Ef1*α*-DIO-EGFP-TVA and AAV-Ef1*α*-DIO-RVG and RV-EnvA-*Δ*G-dsRed were starter cells and can transsynaptic tracing the upstream regions (Figure 6(b)). We can observe the yellow starter cells in LH (Figure 6(c)) and red axon terminal in vPAG (Figure 6(d)); meanwhile, in lots of red RV-infected cells in MPOA, the immunostaining result showed that the RV cells were CaMKII*α*^+^ (Figure 6(e)), which proved that LH CaMKII*α*^+^ cells, which project to vPAG, receive the input from MPOA CaMKII*α*^+^ neurons. We next proved that whether MPOA-LH-vPAG are functionally connected, AAV-hSyn-DIO-ChrimsonR-mCherry was expressed in MPOA of *CaMKIIα-cre* mice (Figure 6(f)) and AAV-Ef1*α*-DIO-axon-GCaMP6s was expressed in LH (Figure 6(g)), and optical cannula were implanted in MPOA for optical activation and in vPAG for fiber photometry (Figure 6(h)). The fluorescent signal of CaMKII*α*^LH-vPAG^axons increased immediately when MPOA CaMKII*α* neurons were activated (Figures 6(i) and 6(j)). The results showed that optically stimulating MPOA CaMKII*α*^+^ neurons induced the activation of LH-vPAG projection, illustrating the functional connection between MPOA-LH-vPAG.

Next, we infected the MPOA with AAV-CaMKII*α*-ChR2-EYFP and the LH with AAV-CaMKII*α*-hM4di-mCherry and then implanted optical cannula in the LH to activate MPOA-LH pathway (Figure S6(a)). Mice were intraperitoneally injected with CNO (1.0 mg/kg) to inhibit LH CaMKII*α*^+^ neurons; interestingly, optogenetic activation of MPOA-LH CaMKII*α* projection can still induce nonappetite hunting behavior (Figure S6(b-d)), which can be due to the retrograde activation of MPOA neuron and induce hunting behavior via MPOA-vPAG pathway. Thus, these results indicate that CaMKII*α*MPOA-vPAG pathway and CaMKII*α* MPOA-LH-vPAG pathway may be compensatory in hunting behavior. Anatomical analysis of the vPAG-projecting LH neurons of sacrificed mice revealed that about 56.60% were GABAergic and that about 40.57% are glutamatergic (Figure S7(a-b)).

Notably, the MPOA-LH-vPAG indirect circuit mediates predatory eating in hunting behavior, while the MPOA-vPAG direct circuit mediates predation but not consumption in hunting behavior (Figure 6(k)).

## 3. Discussion

LH has long been considered as a nucleus controlling feeding behavior. In this research, we revealed a new function of LH CaMKII*α*^+^ neurons in integrating novelty-seeking signal and promoting hunting behavior. When mice are introduced to unfamiliar circumstance or novel object, they display a characteristic pattern of exploration to acquire useful information, such as foraging or discovering escape routes or establish contact with conspecifics. Meanwhile, object retrieval in animals is apparent in situations of food hoarding, nest building, and pup retrieval or has no obvious utility for animals at all [19]. Our results reveal that LH CaMKII*α*^+^ neurons are recruited in novelty-seeking and object retrieval events. To further explore the physiological significance, we find that LH CaMKII*α*^+^ neurons are also activated during hunting; optogenetic stimulation of LH CaMKII*α*^+^ neurons promotes searching, chasing, biting, object retrieval, and feeding behaviors, which behave like hunting; further, LH CaMKII*α*^+^ neurons are proved to sufficiently and necessarily modulate predatory hunting toward artificial and live prey, demonstrating that the novelty-seeking indicates foraging and object retrieval represents food hoarding. We deciphered LH CaMKII*α*^+^ neurons as a key hub to process novelty-seeking signal and convert into hunting action. Attack which happens in conspecies aiming to get mating, territory or social rank is social aggression behavior; happens in interspecies aiming to obtain food is hunting behavior, our results show that LH CaMKII*α*^+^ neurons activation induces attack toward nonsocial object rather than conspecies, demonstrating that LH CaMKII*α*^+^ neurons mediate hunting rather than social aggression behavior.

Furthermore, we deciphered the downstream neural circuit of LH. The PAG is a critical gate for hunting behavior. Lesion of the lateral PAG in mice impairs their ability to chase or attack the prey [38]; what is more, PAG^vGat^ neurons are identified recruited to support the prey search, chase, and attack, but not eating, while PAG^vGluT2^ neurons are only recruited to support the attack during hunting, PAG neuronal ensembles encode sequential hunting motor actions, and this sequential pattern is critically regulated by upstream input signals [39]. Many brain regions including the central amygdala [10], lateral hypothalamus [11], zona incerta [9], and medial preoptical area [13] project to the PAG that mediate hunting behavior. Here, our results showed that LH CaMKII*α*^+^ neurons encode object biting and dislocation; however, CaMKII*α*^LH-vPAG^ projection activation provokes only object biting, and mice stay still and concentrate on biting object rather than retrieve the object. Previous results demonstrated that the central amygdala independently projects to the reticular formation controlling biting attacks and projects to the PAG controlling locomotion during pursuit [10]. We assume that CaMKII*α*^LH-vPAG^ controls maxillofacial muscles; meanwhile, there may be another pathway control locomotion to dislocate object, which remains to be explored. The projection from the LH and CeA may target different subpopulations of the vPAG that both provide excitatory inputs and ultimately lead to a coordinated behavioral output. What is more, LH CaMKII*α*^+^ neuron activation produced nonselective feeding behavior toward edible and inedible objects, but CaMKII*α*^LH-vPAG^ activated mice discriminate caloric food from inedible objects and drive a strong motivation to obtain the food supply and encourage hunting, demonstrating that this circuit plays an important role in identifying caloric food. However, there was no control for retrograde activation of the LH CaMKII*α*^+^ projecting neurons; since mice showed different behavior phenotypes when activating LH CaMKII*α*^+^ soma and CaMKII*α*^LH-vPAG^ projection, we suspect that the observed effect may depend on the activation of the projecting fibers rather than the retrograde activation of LH soma; second, as shown in Figure S(6), activating CaMKII*α*^MPOA-LH^ projection meanwhile inhibits LH CaMKII*α*^+^ neurons, mice still exhibit hunting behavior, and this may due to fiber activation which will also induce LH soma activation. However, whether activation of neural fibers will indeed retrograde activate soma remains to be further explored.

Interestingly, vPAG-projecting LH GABAergic neurons [11] and vPAG-projecting LH Gad2^+^ GABAergic neurons [12] encode predatory hunting but not consumption behavior. While LH CaMKII*α*^+^ neurons promote food pellet feeding and consumption after hunting, immunohistochemistry results showed nonoverlapping of GABAergic neurons and CaMKII*α*^+^ neurons, and the CT-B retrograde tracing from vPAG showed that vPAG receives both GABAergic and CaMKII*α* neuron projections. They are two discrete projections that mediate different types of hunting: GABA^LH-vPAG^ mediates nonappetitive hunting and CaMKII*α*^LH-vPAG^ mediates appetitive hunting. Previous research reported that vPAG-projecting LH glutamatergic neurons encode prediction in danger and evasion [11]. Recent studies elucidated different functions of the LH glutamatergic neurons including promotion of defensive behaviors [40] and inhibition of food intake [32, 41]. These findings seem somewhat contradictory to the finding here that activating the LH CaMKII*α*^+^ neurons, partly glutamatergic neurons, induces feeding, approaching, and hunting behavior; this may be due to the existing highly heterogeneous LH glutamatergic neurons: our results showed that 96.71% vGluT2^+^ neurons are CaMKII*α*^+^ but only 63.87% CaMKII*α*^+^ neurons are vGluT2^+^; remaining 36.13% CaMKII*α*^+^ neurons may be an important part. Interestingly, we found that well-fed mice whose LH vGluT2^+^ neurons were killed exhibited spontaneous hunting behavior toward crickets without optical activation, and optically activating LH vGluT2^−^ CaMKII*α*^+^ neurons induced more robust hunting behavior, indicating that LH CaMKII*α*^+^ vGluT2^−^ neurons are indeed responsible for the promotion of hunting and feeding behavior. What is more, mice perform only hunting behavior and no evasion resulted from the stimulation of all LH CaMKII*α*^+^ neurons; stimulating a large proportion of LH vGluT2^+^, there is no sign of evasion but the 36.13% CaMKII*α*^+^ neurons dominate the behavior phenotype of mice, stating the importance of the small proportion of neurons. Nevertheless, how this small proportion of CaMKII*α*^+^ neurons produce a powerful strength to overcome the drive to evade deserve to be further studied.

Predation is an appetite-driven behavior [1], and previous study revealed the effect of the CaMKII*α*^MPOA-vPAG^ circuiton object exploration and hunting, where this circuit strongly supports the theory that there is a continuum between object exploration and hunting behavior. Nevertheless, it pays little attention to the function of MPOA CaMKII*α*^+^ neurons themselves. Meanwhile, this CaMKII*α*^MPOA-vPAG^ circuit mediates only hunting behavior, and mice will predate the prey but will not consume it, suggesting that there must be another nucleus that receives and integrates the input of novelty-seeking signal into hunting information and mediates following hunting and consuming behavior to satisfy internal energy demand. To replenish the theory, we thoroughly explored the role of MPOA CaMKII*α*^+^ neurons in promoting object exploration and hunting. We found that MPOA send projections across the whole brain and LH receives abundant projection. CaMKII*α*^MPOA-LH^ circuit is also sufficient to promote object exploration and hunting. Our results deciphered that LH CaMKII*α*^+^ neurons receive novelty-seeking signal and hunting information from MPOA and convert into appetite to promote hunting behavior to get energy supply and maintain the homeostasis.

In summary, our study highlights the role of the MPOA-LH-vPAG indirect circuit in object exploration and predatory eating in hunting behavior. This supports the role of the LH CaMKII*α*^+^ neurons as hub that can integrate prey-associated novelty-seeking information and encode motivational hunting and feeding. Our research may enrich the neuronal substrate of hunting behavior, thus providing a better understanding of animal innate behavior.

## 4. Materials and Methods

### 4.1. Animals

Male C57BL/6J mice were purchased from SLAC Laboratory Animal; *CaMKIIα*-ires-*cre* mice (B6 background, stock number: 005359), vesicular GABA transporter (vGaT) Cre-recombinase mice (*Vgat-*ires*-cre*, B6 background, stock number: 016962), vesicular glutamate transporter 2 (vGluT2) Cre-recombinase mice (*vGluT2-ires-Cre*, B6 background, stock number: 016963), and *Ai47* mice (presented by Qi Zilong Laboratory, the cassette containing Emerald-GFP, Tag GFP2, and humanized Renilla-GFP expressed in a Cre-dependent manner) were genotyped according to the protocols provided by the Jackson Laboratory. Female C57BL6/J mice, aged 8–10 weeks, were used in sexual behavior tests. Animals were maintained under a 12 h light/dark cycle with *ad libitum* access to food and water except for the food deprivation experiment. Behavioral experiments were conducted 4 weeks after injection of the viral expression constructs and surgery. All behavioral experiments were conducted between 9:00 and 17:00. All experiments were approved by the Zhejiang University Animal Experimentation Committee and were in complete compliance with the National Institutes of Health Guide for the Care and Use of Laboratory Animals.

### 4.2. Virus Vector

For fluorometric monitoring of soma, AAV-CaMKII*α*-GCaMP6s (serotype: AAV2/9, viral titers: 2.05 × 10^12^ vg/mL, 0.1 *μ*L, OBiO Technology Corp., Ltd. (Shanghai, China)) was injected into C57BL/6J mice. AAV-EF1*α*-DIO-GCaMP6s (serotype: AAV2/9, viral titers: 1.57 × 10^12^ vg/mL, 0.1 *μ*L, OBiO Technology Corp., Ltd. (Shanghai)) was injected into *CaMKIIα-cre* mice. AAV-CaMKII*α*-DO-GCaMP6s (serotype: AAV2/9, viral titers: 5.28 × 10^12^ vg/mL, 0.1 *μ*L, Brain VTA(Wuhan) Corp., Ltd.) was injected into *vGluT2-cre* mice. For fluorometric monitoring of axon, AAV-Ef1*α*-DIO-axon-GCaMP6s (serotype: AAV2/9, viral titers: 3.58 × 10^12^ vg/mL, 0.1 *μ*L, Brain VTA(Wuhan) Corp., Ltd.) was injected into *CaMKIIα-cre* mice. For optogenetic manipulation experiment, AAV-CaMKII*α*-hChR2(H134R)-EYFP (serotype: AAV2/8, viral titers: 1.7 × 10^13^ vg/mL, 0.1 *μ*L, OBiO Technology Corp., Ltd. (Shanghai)) and AAV-CaMKII*α*-ArchT-EGFP (serotype: AAV2/8, viral titers: 1.61 × 10^13^ vg/mL, 0.1 *μ*L, OBiO Technology Corp., Ltd. (Shanghai)) were injected into the MPOA of WT mice. AAV-hSyn-DIO-ChrimsonR-mCherry (serotype: AAV2/9, viral titers: 2.57 × 10^12^ vg/mL, 0.1 *μ*L, Brain VTA(Wuhan) Corp., Ltd.) was injected into *CaMKIIα-cre* mice. For chemogenetic inhibition of LH CaMKII*α*^+^ neurons, AAV-CaMKII*α*-hM4d(Gi)-mCherry (serotype: AAV2/8, viral titers: 1.4 × 10^13^ vg/mL, 0.1 *μ*L, OBiO Technology Corp., Ltd. (Shanghai)) was injected into the LH of WT mice. In retrograde tracing experiments, retrograde tracing Cholera Toxin Subunit B conjugated to Alexa Fluor 647 fluorophore (CT-B 647, titers: 1.0 mg/mL, 0.08 *μ*L, Thermo Fisher Scientific) was used to reveal the direct projections from the MPOA to LH. For inducing neuronal apoptosis, AAV-CMV-DIO-casepase3-TEVp-WPRE virus (serotype: AAV2/9, viral titers: 4.37 × 10^12^ vg/mL, 0.1 *μ*L, Brain VTA(Wuhan) Corp., Ltd.) was injected into *vGluT2-cre* mice.

### 4.3. Stereotactic Virus Injection and Fiber Implantation Surgery

Mice were anesthetized with sodium pentobarbital (50 mg/kg, Sigma-Aldrich) via the intraperitoneal with their head fixed in a stereotaxic apparatus (512600, Stoelting, USA). We made an incision in the mice heads to expose the skull surface and scraped the pericranium away, and burr holes were stereotactically made on the skull. For virus delivery, microinjection was executed using the following coordinates for the nucleus: MPOA (AP, +0.3 mm; ML, +0.2 mm; DV, -5.3 mm, from the bregma), LH (AP, -1.3 mm; ML, ±1.0 mm; DV, -5.0 mm, from the bregma), and vPAG (AP, -4.2 mm; ML, -0.5 mm; DV, -2.3 mm, from the bregma). All of the above coordinates were measured in light of the Paxinos and Franklin's Mice Brain Atlas [42]. A tiny glass capillary connected to 1 *μ*L microliter syringes (Gaoge Industrial and Trading Co. Ltd., Shanghai, China) that was mounted on an Ultra Micro Pump (160494 F10E, World Precision Instruments, USA, WPI) was slowly lowered to the target sites. Virus at a total volume of 0.1 *μ*L was delivered at a flow rate of 20 nL/min at the titers described above. The glass capillary remained in position for another 5 min after injection to allow the diffusion of virus particles and minimize the reflux along the injection track. The virus was allowed to express for a minimum of 4 weeks for adequate accumulation in the soma and axons. CT-B 647 were injected at a total volume of 0.08 *μ*L, and we waited for 1 week for the retrograde tracing to upstream neurons.

For optogenetic manipulation of soma and axons, we implanted optical fiber cannula in the MPOA or LH or vPAG using the coordinates the same as described before, but the depth was 0.2 mm above the virus injection position for enough light delivery. Four screws were implanted in the skull to secure the dental cement. Animals recovered for one week before the behavior test. After the behavioral studies, all virus and optical fiber cannula positions were immunohistochemically confirmed on cryogenic brain sections at the end of all experiments. Only the mice with correct immunohistochemical localization were taken into analysis.

### 4.4. In Vivo Optical Stimulation

Laser light was delivered through a 200 *μ*m diameter optic fiber connected to the laser (blue: Aurora-220, Hangzhou Newdoon Technology Co., Ltd.; yellow: Inper (Hangzhou) Technology Corp., Ltd.). For AAV-CaMKII*α*-ChR2-EYFP activation experiments, blue laser pulses (power 3-5 mW, frequency 20 Hz, pulse width 10 ms) were delivered into the mouse brain. For AAV-hSyn-DIO-ChrimsonR-mCherry activation, a 589 nm yellow laser pulse (power 3-5 mW, frequency 20 Hz, pulse width 10 ms) was delivered. The inhibition experiments used continuous 594 nm yellow light (3-5 mW, direct current).

### 4.5. Fiber Photometry

To record neuronal fluorescence signals, the AAV-CaMKII*α*-GCaMP6s virus was infused into the MPOA or LH as described above. After virus expression for three weeks, an optic fiber (0.20 mm o.d., NA = 0.37; Inper (Hangzhou) Technology Corp., Ltd.) placed in a ceramic ferrule was implanted into either the MPOA, LH, or vPAG. Mice were handled for 5 days to alleviate anxiety evoked by experimenters. The fiber photometry recording system (Thinker Tech) was used. For object exploration behavior, mice were put into the arena for 30 min to acclimate them to the setup. A 3 min baseline was recorded, and a new object was gently delivered into the cage and recorded the real-time calcium signal. For hunting behavior, mice were food-restricted for 12 h then habituated in the environment for 30 min and were recorded for a baseline for 3 min. Then, a cricket was put in the box and the calcium signal was recorded in parallel to a video record during mice hunted. Photometry data were exported into MATLAB for data analysis. *Δ*F/F was calculated as (*F*–*F*_mean_)/*F*_mean_, of which *F*_mean_ was the average fluorescence strength over the baseline period. Fluorescence intensity was calculated as the total photon count over 100 ms. Δ*F*/*F* values for each mouse were presented as heat maps, and the averaged values were presented in plots with the SEM indicated by a shaded area.

### 4.6. Chemogenetic Manipulation

For chemogenetic inhibition, all mice were infected with AAV-CaMKII*α*-hM4d(Gi)-mCherry. Mice were randomly divided into an experimental group and a control group. The experimental group was intraperitoneally injected with clozapine-n-oxide (CNO) (1.0 mg/kg, i.p., Abcam, ab141704), and the control group was intraperitoneally injected with normal saline; mice were performed behavioral test 15 min after the injection.

### 4.7. Behavior Test

All behavioral tests were performed during 9:00-17:00; mice were handled for 10 min for 5 days to reduce fear and anxiety. We conducted most behavior tests in a rectangular transparent arena (30 × 20 × 30 cm). Behavioral tests and analysis were conducted in a blinded fashion, with the experimenter having no idea of the experimental and control groups. Two testers repeated the same experiment to avoid bias. These randomly collected data were sent to an analyst blind to their treatment information. If the same mouse was used to conduct two or more experiments, mouse must rest for at least 3 days between experiments.

### 4.8. Object Exploration

All mice were put into the arena for 20 min each day 5 days before the behavior test to reduce the anxiety. Mice were connected to an optical fiber and habituated to the test chamber 20 min before the formal experiment on test day. After that, a red Styrofoam cube was slightly placed at the center of the arena. We immediately recorded the animal activity in basal state for 3 min, and then 473 nm laser (Newton) was used to optically stimulate the MPOA soma and axon to the LH and vPAG for 3 min; after that, we recorded another 3 min postlight behavior. Mouse tracks were monitored by video recording.

### 4.9. Open Field Test

Mice were placed in the center of a polystyrene enclosure (40 × 40 × 40 cm) and allowed to move freely. The Styrofoam cube was put gently into the center of the arena, the test paradigm was 3 min before light, 3 min light, and 3 min after light. Each stimulation was videotaped individually. The center was defined as the centric 20 × 20 cm area. The open field was cleaned with 70% ethanol between each trial and the track was analyzed using Anymaze software (version 5.29, Stoelting, USA). Total distance traveled (cm) and average velocity (m/s) were analyzed to measure the locomotion. Time in the center (s) and center entries were analyzed to measure the anxiety level.

### 4.10. Chasing Experiment

The experiment was conducted in an open field chamber (40 × 40 × 40 cm) with the outline of the letters “C” and “L” written on the bottom of the chamber. Mice were acclimated to the chamber for 30 min without light. A stick was connected to a red ping-pong ball to guide the mice to chase after it. When mice moved to the left side of the chamber, light (473 nm, 5 mW, 10 ms, 20 Hz) was delivered to the mouse brain constantly. Meanwhile, the ping-pong ball was moved to the start of the letter “C” and was moved to along the “CL” tracks; its speed was modulated to prevent the mouse from touching it. After the mice completed the “L” track, optical stimulation was withdrawn. Control experimental design was performed by the letter “C” with light delivery but the letter “L” without light. Each mouse performed three trials.

### 4.11. Biting Experiment

0.5 g of cotton was kneaded into a tight cotton ball; mice were put in the box to habituate for 30 min. Then, the cotton ball was gently introduced into the box; 3 min baseline animal activities without light were recorded. After that, light was delivered for 3 min and another 3 min was recorded after light. Videos were recorded to analyze the behavior.

### 4.12. Feeding

In electric lesion MPOA experiment, common diet was removed 12 h before the test, and mice had free access to water. In optogenetic activation of MPOA and LH CaMKII*α*^+^ neurons, mice had normal access to food and water. Mice were acclimated to a standard isolation cage (30 × 20 × 30 cm) without bedding for 30 min. A food pellet was introduced into the cage. Mice were allowed to feed for 3 min, and the remaining food pellets and residue were weighed on an electronic scale to measure the weight that mice had consumed.

### 4.13. Social Aggression Test

Aggression was observed using a resident-intruder assay. In this assay, male mice were single-raised for one week to increase motivation for social interactions. Then, they were briefly exposed to a female to increase aggression. On the test day, the female was removed after 1 h, and a younger strange male intruder (20.4–23.0 g) and a new object were introduced into the older resident male (27.0–30.4 g) for 9 min at the same time. After a 3 min light-off session, optical stimulation was delivered for 3 min (light-on; 3 mW, 20 Hz, 10 ms) followed by a 3 min light-off session. The investigation index was calculated as follows: (object investigation time − male investigation time)/(object investigation time + male investigation time).

### 4.14. Object versus Female Competition

In this assay, male mice were single-housed for at least one week to increase motivation for social interactions. Male mice were adapted to the experimental chamber (30 × 20 × 30 cm) for 30 min and then simultaneously exposed to both a female in estrus (C57BL/6J) and an object. After the 3 min free exploration toward female and object, male mice were exposed to 3 min of optical stimulation, after which stimulation was discontinued for 3 min. Time mice spent on the female and object was calculated, respectively. The investigation index was calculated as follows: (object investigation time − female investigation time)/(object investigation time + female investigation time).

### 4.15. Artificial Prey Test

An electrical plastic artificial prey that moved promptly could avoid obstacles, and lacked any caloric value was used. Mice were introduced into the transparent arena (30 × 20 × 30 cm) and adapted for 30 min. The artificial prey was turned on and ran randomly in the box. A video was recorded to analyze mouse behavior.

### 4.16. Hunting Test

Cricket predation experiments were conducted in a transparent, empty, rectangular arena (30 × 20 × 30 cm). Mice habituated the cage for 20 min. 10 min before the optical stimulation, the cage was cleaned again at the beginning of each trial. For optogenetic activation experiments, cricket-naïve and well-fed mice were given adult crickets of medium size (15-20 mm length). A 473 nm blue light (3-5 mW, 10 ms, 20 Hz, 30 s on, 10 s off) was delivered for 5 min. For attack possibility experiments, 5 crickets of medium size (15-20 mm length) were introduced into the cage, a 473 nm blue light (3-5 mW, 10 ms, 20 Hz, 30 s on,10 s off) was delivered for 5 min, the number of crickets that were attacked was counted, and the proportion of attacked crickets were calculated after that.

In order to guarantee starving mice to perform reliable predatory hunting behavior, it was necessary to acclimate the mice to the crickets. Firstly, mice were acclimated to the experimenter and fed crickets once per day in their home cage for 3 days to alleviate the fear of crickets. After 3 days of capture trials, all of the mice captured prey reliably. Capture performance was deemed reliable if three sequential trials each ended in cricket capture in under 30 s. Then, following 12 h of food deprivation, mice were introduced to the arena for 30 min to habituate to the setup. Then a cricket of medium size (15-20 length) was put into the arena and a 589 nm direct current (3-5 mW, 50 ms, 20 Hz, 5 min) was delivered.

For chemogenetic inhibition of LH, mice were food-deprived for 12 h. On the test day, mice were intraperitoneally injected with CNO or normal saline. 15 min later, the hunting experiment began. For the attack possibility experiments, 5 crickets of medium size (15-20 mm length) were introduced into the cage for 5 min, and the number of attacked crickets was calculated. Other hunting-related parameters were also measured as follows: latency to hunt: time taken from optical stimulation onset until a mouse chased a cricket; hunting duration: time elapsed from mice starting pursing the prey until successfully capturing the crickets using two forepaws or mouth, not necessarily killing the crickets; cricket eating duration: time taken from mice capturing the crickets until mice stopped chowing the crickets; and attack possibility: quantified as the percentage of the total crickets that are attacked.

### 4.17. Histological Examination

After all behavior experiments were performed, mice were sacrificed and perfused intracardially with iced phosphate buffer (PBS) followed by 4% formaldehyde in 0.1 M PBS, and brains were removed and postfixed with 4% formaldehyde overnight, followed by dehydration in 30% sucrose at 4°C for two days. Coronal sections were cut on a cryotome (Thermo Fisher Scientific, Cryostar NX70, USA). For virus and optical fiber cannula position post hoc tests, coronal sections (30 *μ*m depth) were repeatedly washed in PBS and sealed with DAPI Fluoromount-GTM (Lot: 36308ES20, Yeasen, China). Images were captured by a virtual digital slice scanning system (VS120, OLYMPUS, Japan). If the optical fiber was located above (within 0.5 mm) the goal region with the correct viral fluorescence expression, the corresponding behavior data was taken into analysis. For virus specificity verification, coronal sections (40 *μ*m depth) were cut and collected into a 24-well plate for immunohistochemical labeling experiments. Brain section was incubated with 0.1% Triton X-100 for 15 min and then incubated with 5% donkey serum albumin (Yeasen Biotech Co., Ltd., CAT: 36119ES10) for 2 h after at room temperature (RT) and was incubated at 4°C with primary antibody overnight. After washing three times for 5 min with PBS, the sections were incubated with a secondary antibody for 2 h at RT. After repeated washing, the sections were then covered with glass coverslips, and images were captured via a confocal microscope (DMi8, Leica, Germany). For the Fos protein mapping experiment, a 473 nm light was delivered in MPOA for 30 min (30 s on, 30 s off). 1.5 h later, the mice were sacrificed for the whole brain immunohistochemical labeling for Fos protein.

Antibodies Used in This Research are Shown in Table 1

### 4.18. Combined Fluorescence In Situ Hybridization

Coronal sections (14 mM) were cut on a freezing microtome (Thermo NX50). The mouse Slc17a6 (Cat#319171) combined with RNAscope 2.5 HD Detection Reagent Kit-Red/RNAscope Multiplex Fluorescent Reagent kit v2 (Advanced Cell Diagnostics) was used. Fluorescent images were taken using Leica SP8 laser confocal microscope.

### 4.19. Statistics

No statistical analyses were performed to predetermine sample sizes, but the sample sizes used were similar to those used in many previous studies that employed proximate neurochemical, electrophysiological, optogenetic, and behavioral techniques [43, 44]. Data were assessed by Student's *t*-test, paired *t*-tests, and one-way ANOVA tests appropriately using GraphPad Prism 7 software (9.0; GraphPad Software). For parameters that followed a normal distribution (Shapiro-Wilk test, *P* > 0.05), differences between two groups were analyzed with the Student's *t*-test, and comparisons of three or more groups were conducted via ANOVA. The Mann-Whitney *U* test and Wilcoxon signed-rank test were used for data that were not normally distributed. Differences in variance were determined in preliminary tests and the Welch corrections were unnecessary. The Holm-Sidak method was used to correct for multiple comparisons. All statistical tests were two-sided, and *P* values < 0.05 were considered statistically significant. All data were expressed as means ± SEMs.

## Figures and Tables

**Table 1:** Antibodies used in this research.

Antibody	Manufacture	Identifier	Reference	PubMed ID	RRID	Application
Anti-CaMKII*α* in rabbit	Abcam	ab134041	Medinaceli Quintela R et al. *J Neurosci*. 40:7269-7285 (2020)	32817250	AB_2811181	1 : 800
Anti-Fos in guinea pig	Synaptic Systems	226308	Bolós M et al. J *Neurosci*. 39 (9):1605-1620 (2019)	30651327	AB_2619946	1 : 2000
Anti-GABA in rabbit	Sigma-Aldrich	A2052	Xu C et al. *Cell*. 167 (4):961-972 (2016)	27773481	AB_477652	1 : 800
Anti-GFP in chicken	Abcam	ab13970	Horita N et al. *Cell Mol Gastroenterol Hepatol* 13:275-287 (2022)	34438113	AB_300798	1 : 1000
Anti-rabbit 594 in donkey	Abcam	ab150064	Mihagric M et al. *Elife* 10:e61170 (2021)	33480356	AB_2734146	1 : 800
Anti-rabbit 488 in goat	Abcam	ab150077	Li Y et al. *Redox Biol*. 38:101771 (2021)	33189984	AB_2651036	1 : 800
Anti-chicken 488 in goat	Abcam	ab150169	Arguello JR et al. *Elife*. 16 (4):784-796 (2021)	33666172	AB_2636803	1 : 800
Anti-guinea pig 647	Abcam	ab150187	Cooler S et al. *Nat Neurosci* 24:105-115 (2021)	33230322	AB_2827756	1 : 800

## Data Availability

All data required to support the conclusions are presented in the main text and the supplementary materials.

## Supplementary Materials

Supplementary Materials Figure S1: verification of the neuronal type of LH CaMKII*α*^+^ neurons. Figure S2: viral-assisted downstream region mapping of MPOA CaMKII*α*^+^ neurons. Figure S3: activation of MPOA CaMKII*α*^+^ neurons initiates novelty-seeking behavior during object exploration. Figure S4: MPOA CaMKII*α*^+^ neurons are critically involved in hunting behavior. Figure S5: activation of CaMKII*α*^MPOA-LH^ circuit induces nonsocial interaction and will not induce social aggression. Figure S6: CaMKII*α*^MPOA-vPAG^ pathway and CaMKII*α*^MPOA-LH-vPAG^ pathway are compensatory in hunting behavior. Figure S7: vPAG receives both GABAergic and glutamatergic projection from the LH. Video S1: in vivo fiber photometry of LH CaMKII*α*^+^ neurons when mice explored novel object and hunting (related to Figures 1 and 2). Video S2: LH CaMKII*α*^+^ neuron activation induces object exploration and object chasing behavior (related to Figure 1). Video S3: different behavioral phenotypes between LH CaMKII*α*^+^ neuron activation and MPOA CaMKII*α*^+^ neuron activation (related to Figure 1 and Figure S4). Video S4: LH CaMKII*α*^+^ neuron activation induces nonselective feeding behavior (related to Figure 1). Video S5: LH CaMKII*α*^+^ neuron activation promotes predatory-like attack toward artificial prey and lively crickets and mice prefer to hunt live prey to readily food pellet (related to Figure 2). Video S6: different behavior phenotypes between LH CaMKII*α*^+^ neuron activation and CaMKII*α*^LH-vPAG^ projection activation (related to Figures 1 and 3). Video S7: optical inhibit MPOA CaMKII*α*^+^ neurons abolish internal appetite-induced hunting behavior (related to Figure S4).
