# Supplementary material for: Lateral Hypothalamus Calcium/Calmodulin-Dependent Protein Kinase II α Neurons Encode Novelty-Seeking Signals to Promote Predatory Eating
Source: Research (Wash D C). 2022 Aug 5;2022:9802382. doi: 10.34133/2022/9802382 (PMC9394055; doi:10.34133/2022/9802382)
Supplement: Supplementary Materials — Figure S1: verification of the neuronal type of LH CaMKIIα+ neurons. Figure S2: viral-assisted downstream region mapping of MPOA CaMKIIα+ neurons. Figure S3: activation of MPOA CaMKIIα+ neurons initiates novelty-seeking behavior during object exploration. Figure S4: MPOA CaMKIIα+ neurons are critically involved in hunting behavior. Figure S5: activation of CaMKIIαMPOA-LH circuit induces nonsocial interaction and will not induce social aggression. Figure S6: CaMKIIαMPOA-vPAG pathway and CaMKIIαMPOA-LH-vPAG pathway are compensatory in hunting behavior. Figure S7: vPAG receives both GABAergic and glutamatergic projection from the LH. Video S1: in vivo fiber photometry of LH CaMKIIα+ neurons when mice explored novel object and hunting (related to Figures 1 and 2). Video S2: LH CaMKIIα+ neuron activation induces object exploration and object chasing behavior (related to Figure 1). Video S3: different behavioral phenotypes between LH CaMKIIα+ neuron activation and MPOA CaMKIIα+ neuron activation (related to Figure 1 and Figure S4). Video S4: LH CaMKIIα+ neuron activation induces nonselective feeding behavior (related to Figure 1). Video S5: LH CaMKIIα+ neuron activation promotes predatory-like attack toward artificial prey and lively crickets and mice prefer to hunt live prey to readily food pellet (related to Figure 2). Video S6: different behavior phenotypes between LH CaMKIIα+ neuron activation and CaMKIIαLH-vPAG projection activation (related to Figures 1 and 3). Video S7: optical inhibit MPOA CaMKIIα+ neurons abolish internal appetite-induced hunting behavior (related to Figure S4). [file 9802382.f1.zip › Tan_SI.docx]

**Lateral Hypothalamus Calcium/calmodulin-dependent protein kinase Ⅱ α Neurons Encode Novelty-Seeking Signals to Promote Predatory Eating**

Na Tan ^1^, Jiaying Shi ^1^, Lingyu Xu ^1^, Yanrong Zheng ^2^, Xia Wang ^1^, Nanxi Lai ^1^, Zhuowen Fang ^1^, Jialu Chen ^1^, Yi Wang ^1, 2^, and Zhong Chen ^1, 2*^

**Affiliations**

*^1^ Institute of Pharmacology and Toxicology,* *College of Pharmaceutical Sciences, Zhejiang University, Hangzhou, China.*

*^2^ Key Laboratory of Neuropharmacology and Translational Medicine of Zhejiang Province, School of Pharmaceutical Sciences, Zhejiang Chinese Medical University, Hangzhou, China.*

Correspondence should be addressed to Zhong Chen; chenzhong@zju.edu.cn

**Supplementary Information**


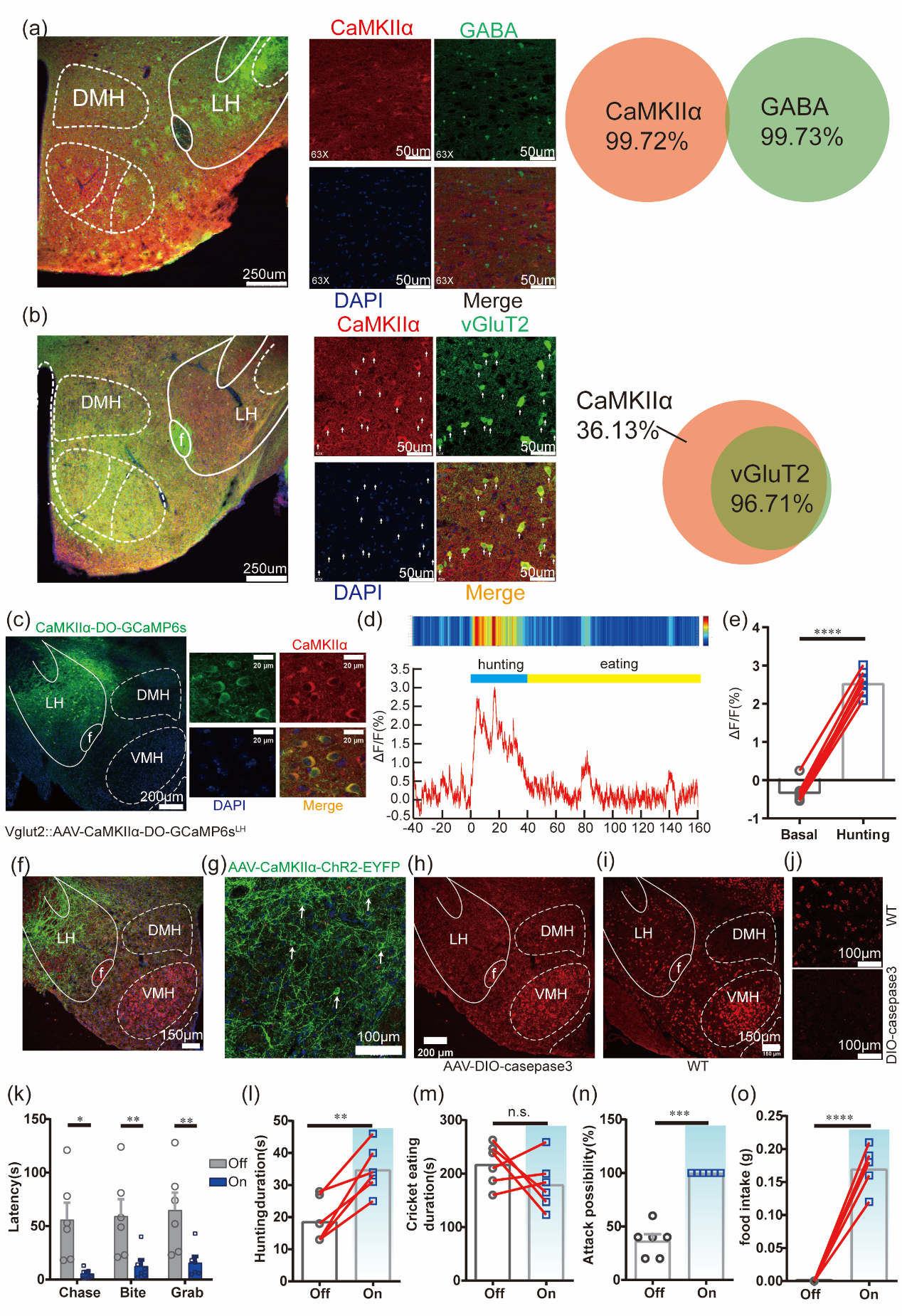
0

**FIGURE S1: Verification of the neuronal type of LH CaMKIIα^+^ neurons** (a) Left, a representative image of the LH region of the cross line of *Vgat-cre* mice and *Ai47* mice, scale bar, 250 µm; middle, a representative image of immunohistochemical labeling of CaMKIIα, scale bar, 50 µm; right, percentage of co-localization the GABAergic neurons and CaMKIIα^+^ neurons. (b) Left, a representative image of LH region of the cross line of *vGluT2-cre* mice and *Ai47* mice, scale bar, 250 μm; middle, a representative image of immunohistochemical labeling of CaMKIIα, scale bar, 50 µm; right, percentage of co-localization of vGluT2^+^ neurons and CaMKIIα^+^ neurons. (c) Left, a representative image of LH infected with AAV-CaMKIIα-DO-GCaMP6s virus, scale bar, 200 µm; right, representative images of co-localization of GCaMP6s expression (green) and immunolabelled CaMKIIα (red) and DAPI (blue), scale bar, 20 μm. (d) Representative raw trace and heat map during hunting (blue) and cricket consumption (yellow) processes. (e) Average GCaMP signal aligned to the start of hunting (each of four mice was repeated for twice, paired t test, *****P* < 0.0001). (f) Representative image of LH infected with AAV-CaMKIIα-ChR2-EYFP and AAV-DIO-casepase3 virus and in suit hybridization of vGluT2 (red), scale bar, 150 µm. (g) Enlarged image of LH, scale bar 100 µm. (h) Representative image of LH with vGluT2 neurons apoptosis, scale bar 200 µm. (i) Representative image of LH of WT mice, scale bar 150 µm. (j) Enlarged images of LH vGluT2 neurons apoptosis and WT mice, Scale bar, 100 µm. (k-n) Effects of optical stimulation of LH CaMKIIα^+^ vGluT2^-^ neurons on hunting behavior of well-fed mice; latency of each procedure when hunting (k), duration of hunting (l); cricket eating duration (m) and attack possibility of 5 crickets (n) (n=6, paired *t*-test, **** *P* <0.0001, *** *P* < 0.001, ***P* < 0.01, * *P* < 0.05). (o) Food intake in three minutes (paired *t*-test, **** *P* <0.*0001*)

**
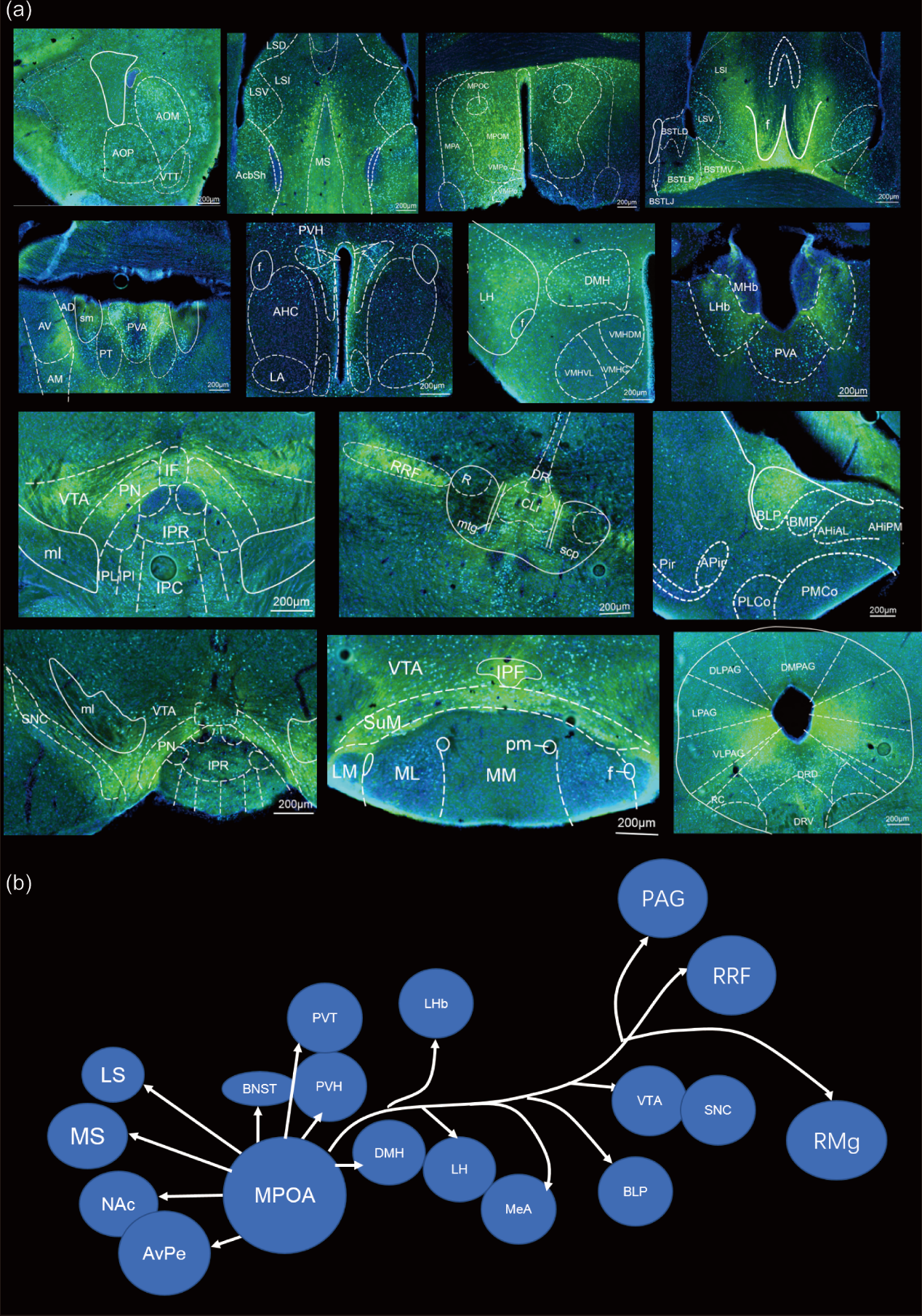
**

**FIGURE S2: Viral-assisted downstream regions mapping of MPOA CaMKIIα^+^ neurons.** (a) The AAV-CaMKIIα-ChR2-EYFP virus was expressed on both the neuronal soma and terminal. By tracking the expression of enhanced yellow fluorescence, the downstream nuclei of the MPOA can be mapped. AAV-CaMKIIα-ChR2-EYFP was injected into the MPOA of C57BL/6J mice and brain sections were prepared. Fluorescence marked the nuclei that MPOA CaMKIIα^+^ neurons sent projections to. After optical stimulation of the MPOA CaMKIIα^+^ neurons for 30 min, whole brain products of Fos protein were mapped, the cyan color represents Fos and the green color represents the projection. (b) Schematic summary of whole brain output targets of MPOA CaMKIIα^+^ neurons.


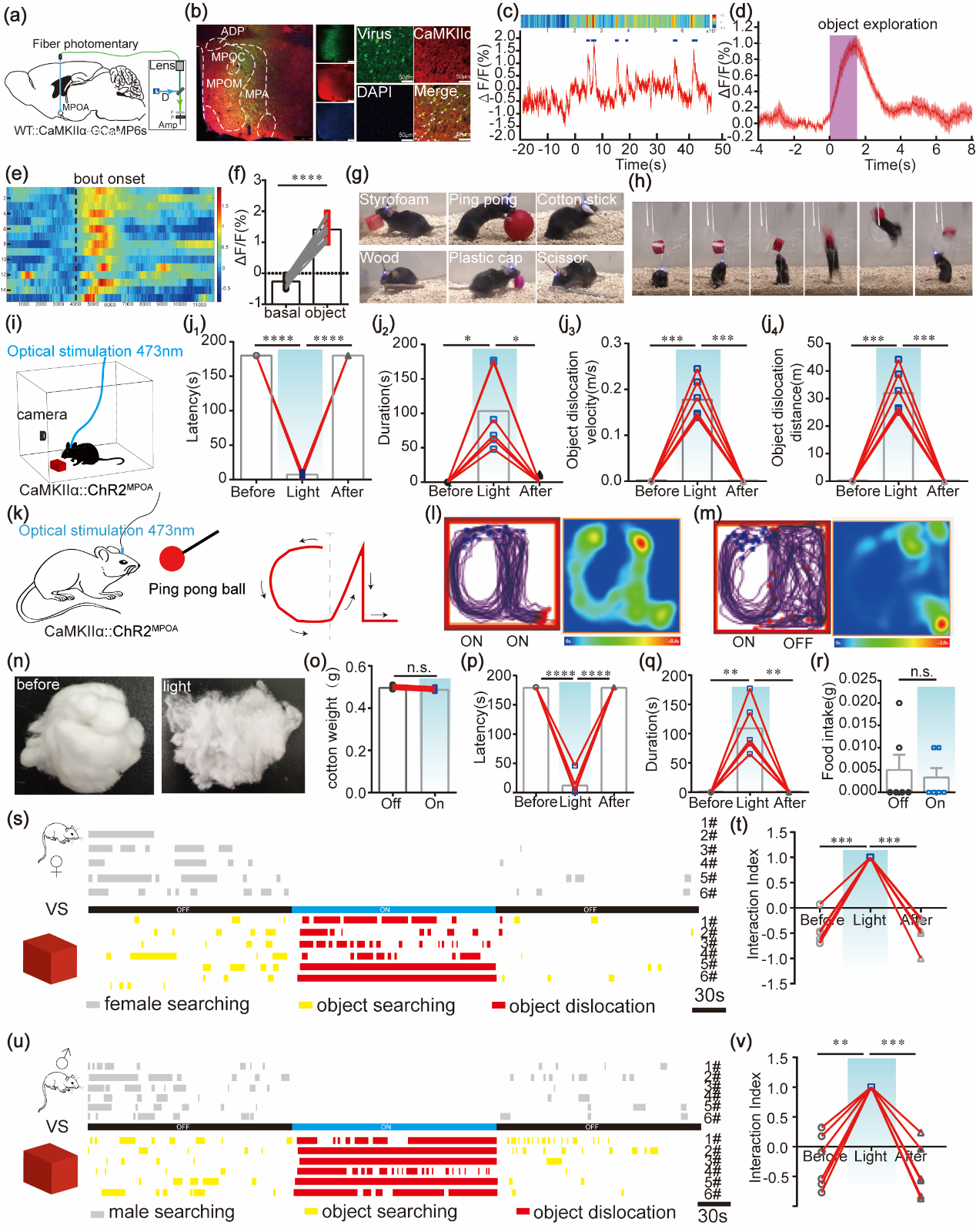


**FIGURE S3: Activation of MPOA CaMKIIα^+^ neurons initiate novelty-seeking behavior during object exploration** (a) Schematic of fiber photometry of neuronal calcium signals in the MPOA CaMKIIα^+^ neurons expressing GCaMP6s from C57BL/6 male mice while exploring objects or hunting crickets. Amp, amplifier; D, dimorphic mirror; F, filter; P, photomultiplier tube. AAV-CaMKIIα-GCaMP6s was injected into the MPOA and fiber-optic cannulas were implanted above it. (b) Left, GCaMP6s expression in MPOA, scale bar, 500 μm; right, co-localization of GCaMP6s expression and immunostaining of CaMKIIα, scale bar, 50 μm. (c) Representative raw calcium signal traces and heat map for object exploration behavior. Blue bars indicated each bout of object exploration. During this experiment, mice contacted the object with its nose for six times. (d) Mean GCaMP signal *(ΔF/F= (F-F_mean_)/ F_mean_)* aligned to the initiation of object exploration from 5 mice during the experiment (each mouse was repeated for three times). (e) Heat map of fluorescence alteration of 5 mice (each mouse was repeated for three times), aligned to bout onset the error bar indicated SEM of all trials. (f) Quantification of fluorescence changes during object exploration compared to the free moving state of 5 mice (each mouse was repeated for three times; paired *t*-test, *****P* < 0.0001). (g) Representative images of CaMKIIα::ChR2^MPOA^ mice retrieving different sized and textured objects. (h) Representative video snapshots of mice showed sequential postures of leaping and jumping used to reach an object hanging in the air. (i) Schematic of mice explored objects during optogenetic modulation. (j) Effects of optical activation of MPOA CaMKIIα^+^ neurons on object exploration behavior. Latency of object exploration (j_1_); duration of object dislocation (j_2_); average object dislocation velocity (j_3_); object dislocation distance (j_4_); (n = 6; one-way ANOVA, followed by Dunnett post-hoc test, **P* < 0.05, ****P* < 0.001, *****P* < 0.0001). (k) Schematic of CaMKIIα::ChR2^MPOA^ mice followed the Ping-pong ball moved along with contours of the letters ‘‘C’’ and ‘‘L’’. (l) The cumulative traces and mean heat map of the mice navigation pathways, light was provided during the whole process; (m) The cumulative traces and mean heat map of the mice navigation pathways, light was withdrawn after finishing the letter “C” (n = 6). (n-q) Effect of optical stimulation of CaMKIIα^MPOA^ neurons on biting a cotton ball. Representative image of a cotton ball from before and after light delivery (n), cotton weight (o), latency of biting (p), duration of biting cotton (q) evoked by MPOA CaMKIIα^+^ activation. (r) Effects of optical activation on feeding behavior (n = 6, paired *t*-test, *P* = 0.6109). (s) Optical activation of CaMKIIα::ChR2^MPOA^ neurons, behavioral raster plots illustrating synchronous interaction of male mice with a Styrofoam cube and a female mouse. Behaviors toward different targets were illustrated individually. (t) Optical stimulation altered the interaction index (n = 6; one-way ANOVA followed by Dunnett post-hoc test, ****P* < 0.001). (u) Optical activation of CaMKIIα::ChR2^MPOA^ neurons, behavioral raster plots illustrating synchronous interaction of male mice with a Styrofoam cube and a male mouse. Behaviors toward different targets were illustrated individually. (v) Optical stimulation changed the interaction index (n = 6; one-way ANOVA, Dunn’s multiple comparisons test, ***P* < 0.01, ****P* < 0.001).


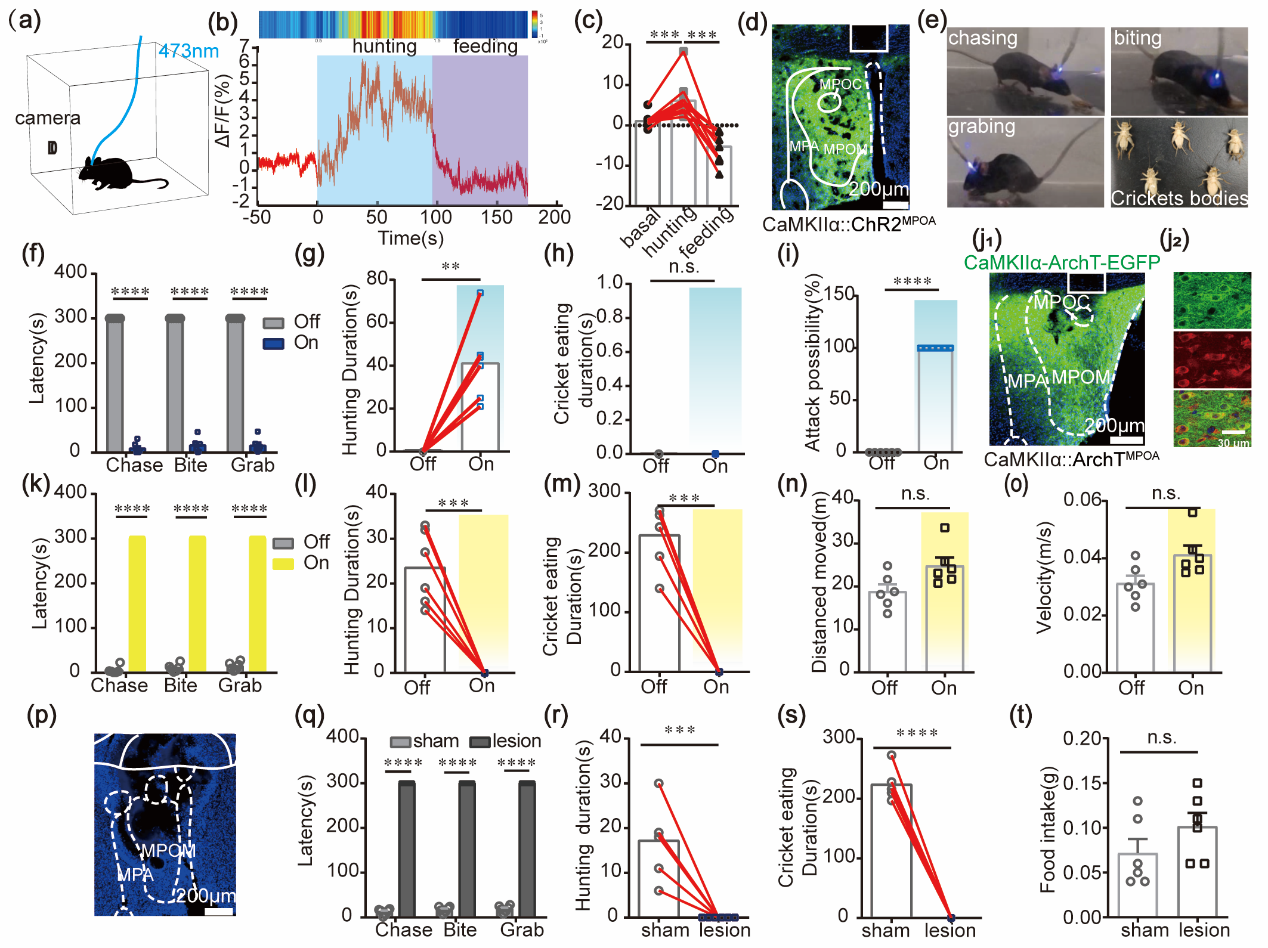


**FIGURE S4: MPOA CaMKIIα^+^ neurons are critically involved in hunting behavior.** (a)Schematic of mice hunting. Mice were connected to the optical fibers simultaneously with video recorded in the box. (b) Representative raw calcium signal trace and heat map for hunting. The green bar indicated the process of hunting and the purple bar indicated the process of consumption. (c) Quantification of fluorescence changes during hunting and consumption of 5 mice (each mouse was repeated for three times; one-way ANOVA followed by Dunnett post-hoc test, ****P* < 0.001). (d-i) Effects of optical activation of MPOA CaMKIIα^+^ neurons on cricket hunting behavior. ChR2 expression and optical fibers cannula position, scale bar, 200 µm (d); Schematic of hunting sequence and the dead bodies of crickets (e); latency of each posture of hunting sequence (f), cricket hunting duration (g), cricket eating duration (h) and the possibility of predatory attack (i) induced by optical activation of the CaMKIIα^+^ MPOA neurons in well-fed CaMKIIα::ChR2^MPOA^ mice (n = 6, paired *t*-test: *****P* < 0.0001; ***P* < 0.01). (j-q) Effects of optical inhibition of MPOA CaMKIIα^+^ neurons on starvation-induced hunting behavior. (j_1_), representative image of ArchT-EGFP expression and optical fibers cannula position, scale bar, 200 µm; (j_2_), co-localization of the AAV- ArchT-EGFP expression (green) and immunostaining of CaMKIIα (red), scale bar, 30 µm. (j); latency of chasing, biting, capturing crickets (K), cricket hunting duration (l) and cricket eating duration (m) induced by photoinhibition (n = 6, paired *t*-test: *****P* < 0.0001, ****P* < 0.001). (n) Distance moved before and during light inhibition (paired *t*-test, *P* = 0.0718); (o) average velocity (paired *t*-test, *P* = 0.0671). (p-t) The effect of MPOA lesion on hunting behavior; p, Representative images of lesion of MPOA of mice, Electrodes (0.125 mm in diameter; A.M. Systems, USA) were implanted to the MPOA (AP, +0.3 mm; ML +0.2 mm, DV –5.3 mm), scale bar, 200 µm, electrical stimulation was delivered (1.5 mA) for 10 s each, mice in sham group implanted electrode but did not receive any electrical stimulation. Latency of sequential action of hunting (q), hunting duration between lesion and sham group (r) and cricket eating duration (s) between lesion and sham group (n = 6, paired *t*-test, *****P* < 0.0001, ****P* < 0.001)**.** (t) food intake between lesion and sham group (n = 6, paired *t*-test, *P* = 0.2557).


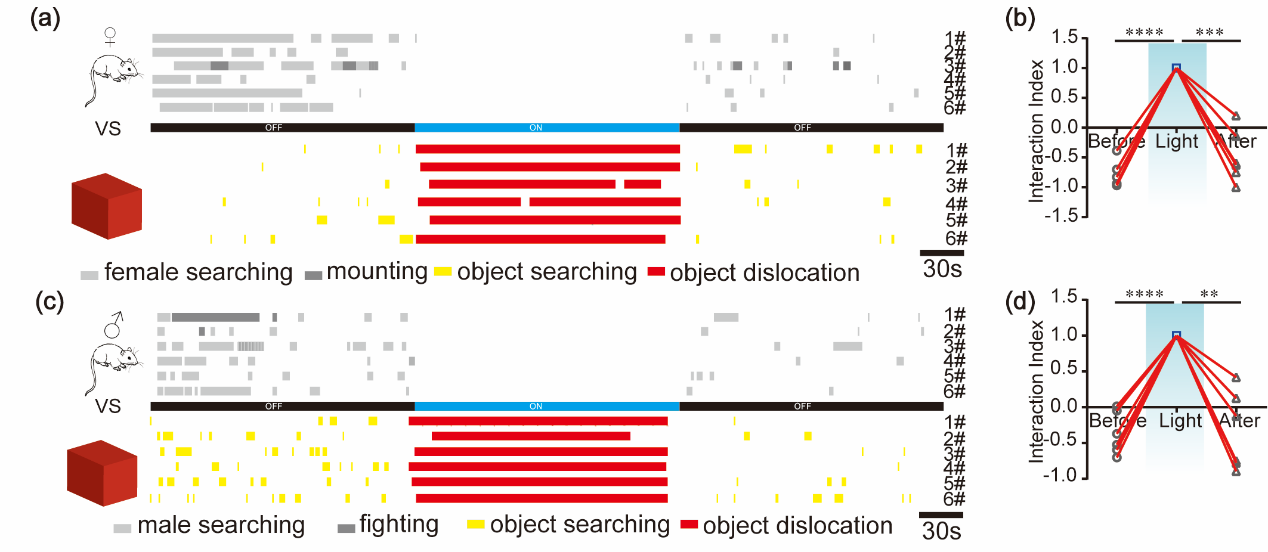


**FIGURE S5: Activation CaMKIIα^MPOA-LH^ circuit induces non-social interaction and will not induce social aggression** (a) Optical activation of the CaMKIIα::ChR2^MPOA-LH^ circuit, behavioral raster plots illustrated synchronous interaction of male mice with a Styrofoam cube and a female mouse. Behaviors toward different targets are illustrated individually. (b) Optical stimulation changed the interaction index (n = 6; one-way ANOVA followed by Dunnett post-hoc test, *****P* < 0.0001, ****P* < 0.001). (c) Behavioral raster plots illustrated activation of CaMKIIα::ChR2^MPOA–LH^ circuit synchronous interaction of male mice with a Styrofoam cube and a younger male mouse. Behaviors toward different targets were illustrated separately. (d) Optical stimulation changed the interaction index (n = 6; one-way ANOVA followed by Dunnett post-hoc test, *****P* < 0.0001, ***P* < 0.01).


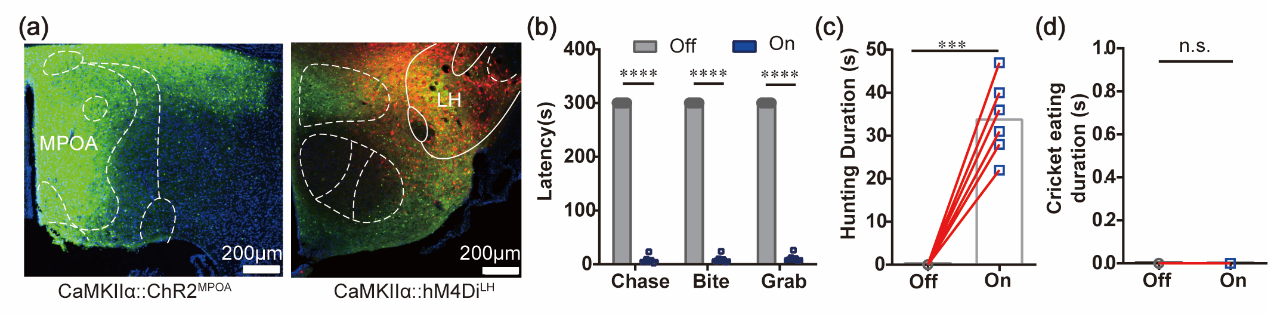


**FIGURE S6** **CaMKIIα^MPOA-vPAG^ pathway and** **CaMKIIα^MPOA-LH-vPAG^ pathway are compensatory in hunting behavior.** (a-d) Effects of optical activation of CaMKIIα^MPOA-LH^ projection, in the presence of chemogenetic inhibition of LH CaMKIIα neurons, on cricket hunting behavior. Representative image ChR2-eYFP expression in MPOA (left) and hM4Di-mcherry in LH (right), scale bar, 200 µm (a); latency of each posture of hunting sequence (b), cricket hunting duration (c), cricket eating duration (d) (n = 6, paired *t*-test: *****P* < 0.0001; ****P* < 0.001).

**
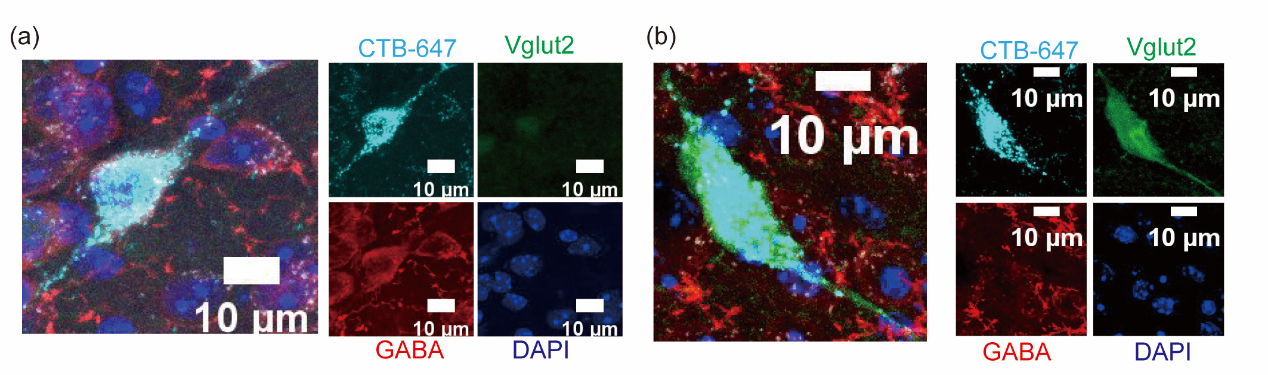
FIGURE S7 vPAG receives both GABAergic and glutamatergic projection from the LH.** (a) Representative images of CTB 647 retrograde soma in the LH and colocalization with immunostaining of GABA (red), scale bar, 10 µm. (b) Representative images of CTB 647 retrograde soma in the LH and colocalization with immunostaining of CaMKIIα (red), scale bar, 15 µm.

**Supplementary Videos**

**Video S1. In vivo fiber photometry of LH CaMKIIα^+^ neurons when mice explored novel object and hunting. (Related to Fig.1 and Fig.2)**

When mice were introduced with novel objects, they would constantly explore it out of instinct, LH CaMKIIα^+^ neurons Ca^2+^ concentration increased immediately when mice explored the object and maintained a high level when mice retrieved it, Ca^2+^ concentration decreased immediately when mice threw down the object (00:00:01-00:00:19). In vivo fiber photometry showed that LH CaMKIIα^+^ neurons activity increased when predated crickets and decreased when consumed the dead body (00:00:20-00:00:50).

**Video S2. LH CaMKIIα^+^ neurons activation induces object exploration and object chasing behavior (Related to Fig.1)**

When an inedible object was placed in the arena, optical activated LH CaMKIIα^+^ neurons induced mice to perform “hunting-like” posture including exploring, biting, and retrieval (00:00:01-00:00:19). If the object was too big to bit, mice would hug the ping-pong ball with two forepaws and attempt to bite it (00:00:20-00:00:38). Optical activation of LH CaMKIIα^+^ neurons induced mice to chase after moving ping-pong ball along designed route, mice moved randomly once the light was withdrawn (00:00:39-00:01:13).

**Video S3. Different behavioral phenotypes between LH CaMKIIα^+^ neurons activation and MPOA CaMKIIα^+^ neurons activation (Related to Fig.1 and Fig.S4)**

Optical activation of LH CaMKIIα^+^ neurons induced mice to dislocate and voraciously consume food pellets (00:00:01-00:00:19); however, mice only dislocated food pellets but not ate food pellets when MPOA CaMKIIα^+^ neurons were optical activated (00:00:20-00:00:47).

**Video S4. LH CaMKIIα^+^ neurons activation induces non-selective feeding behavior (Related to Fig.1)**

Mice dislocated and bit edible and inedible objects when optical activated LH CaMKIIα^+^ neurons. LH CaMKIIα^+^ neurons activation abolished the natural preference for caloric food and promoted compulsively feeding behavior (00:00:01-00:00:19).

**Video S5. LH CaMKIIα^+^ neurons activation promotes predatory-like attack toward artificial prey and lively crickets and mice prefer to hunt live prey to readily food pellet (Related to Fig.2)**

When mice were introduced to an annoying moving artificial prey, optical activation of LH led mice to pursue, bite, and restrain the artificial prey (00:00:01-00:00:17). Optical activated LH CaMKIIα^+^ neurons induced stretching of the neck and the release of biting attacks, followed by biting and prey restraint using the forepaw and followed by dead body consuming (00:00:18-00:00:43). Compared with readily caloric food pellets, mice preferred to predate live crickets when LH CaMKIIα^+^ neurons were activated (00:00:44-00:01:08).

**Video S6. Different behavior phenotypes between LH CaMKIIα^+^ neurons activation and** **CaMKIIα^LH-vPAG^ projection activation (Related to Fig.1 and Fig. 3)**

Mice would chase inedible moving object when LH CaMKIIα^+^ neurons were activated (00:00:01-00:00:21); however, mice preferred to chase caloric food pellet when optical activated CaMKIIα^LH-vPAG^ projection (00:00:22-00:00:39).

**Video S7. Optical inhibit MPOA CaMKIIα^+^ neurons abolish internal appetite-induced hunting behavior (Related to Fig.S4)**

The hunting process was immediately interrupted when MPOA CaMKIIα^+^ neurons were optical inhibited of food-deprived mice, mice resumed predating cricket when optical inhibition was withdrawn (00:00:01-00:00:28).
